# Identification and verification of an ALYREF-involved 5-methylcytosine based signature for stratification of prostate cancer patients and prediction of clinical outcome and response to therapies

**DOI:** 10.1007/s12672-023-00671-w

**Published:** 2023-05-08

**Authors:** Xiao Tan, Zhouda Cai, Guo Chen, Chao Cai, Jiahong Chen, Yingke Liang, Yangjia Zhuo, Jianming Liu, Liangliang Huang, Bin Ouyang, Yanni Wei, Zhenyu Jia, Junhong Deng, Weide Zhong, Jianming Lu

**Affiliations:** 1 Department of Andrology, Guangzhou First People’s Hospital, Guangzhou Medical University, Guangzhou, 510180 China; 2 Department of Urology, Guangdong Key Laboratory of Clinical Molecular Medicine and Diagnostics, Guangzhou First People’s Hospital, Guangzhou Medical University, Guangzhou, 510180 China; 3 Department of Urology, School of Clinical Medicine, The Affiliated Hospital of Southwest Medical University, Southwest Medical University, Luzhou, 646000 Sichuan China; 4 Department of Urology, The First Affiliated Hospital of Jinan University, Guangzhou, Guangdong China; 5 Department of Urology, Minimally Invasive Surgery Center, The First Affiliated Hospital of Guangzhou Medical University, Guangdong Key Laboratory of Urology, Guangzhou Institute of Urology, Guangzhou, 510120 Guangdong China; 6 Department of Urology, Huizhou Municipal Central Hospital, Huizhou, 516001 Guangdong China; 7 Department of Botany and Plant Sciences, University of California, Riverside, CA 92521 USA; 8 Macau Institute for Applied Research in Medicine and Health, Macau University of Science and Technology, Macau, China

**Keywords:** Prostate cancer, Epigenetics, RNA 5-methylcytosine, Biochemical recurrence, Androgen receptor signaling inhibitor, Immunotherapy

## Abstract

**Objectives:**

Due to the heterogeneity of PCa, the clinical indicators used for PCa can't satisfy risk prognostication and personalized treatment. It is imperative to develop novel biomarkers for prognosis prediction and therapy response in PCa. Accumulating evidence shows that non-mutational epigenetic reprogramming, independent from genomic instability and mutation, serves as a newly added hallmark in cancer progression.

**Methods:**

In this study, we integrated multi-center cohorts (N > 1300) to develop a RNA 5-methylcytosine regulator-based signature, the m5C score. We performed unsupervised clustering and LASSO regression to identify novel m5C-related subtypes and calculate the m5C score. Then we assessed the role of m5C cluster and m5C score in several clinical aspects such as prognosis in various molecular subtypes, responses to chemotherapy, androgen receptor signaling inhibitor (ARSI) therapy and immunotherapy in PCa. Finally, we validated the cancer-promoting performance of ALYREF through clinical data analysis and experiments in vivo and in vitro.

**Results:**

The investigation revealed that the m5C score could accurately predict the biochemical recurrence (BCR) in different subtypes (the PAM50 subtypes and immunophenotypes) and the responses to chemotherapy, ARSI therapy, and immunotherapy (PD1/PD-L1). A high m5C score indicated a poor BCR prognosis in every subtype of PCa, unfavorable responses in ARSI therapy and immunotherapy (PD1/PD-L1). Moreover, the m5C reader gene termed *ALYREF,* yielding the highest weighed coefficient, promoted PCa progression through in silico analysis and experimental validations (in vivo and in vitro).

**Conclusions:**

The m5C signature can function in many aspects of PCa, such as the development and prognosis of the disease, and multiple therapy responses. Further, the m5C reader, ALYREF, was identified as a prognostic biomarker and a potential therapeutic target for PCa. The m5C signature could act as a brand-new tool for predicting the prognosis of patients in different molecular subtypes and patients’ therapy responses and promoting customized treatments.

**Supplementary Information:**

The online version contains supplementary material available at 10.1007/s12672-023-00671-w.

## Introduction

Currently, prostate cancer (PCa) is the second most common malignant cancer diagnosed in men worldwide. It is the 5th leading cause of cancer-specific death in male patients, with deaths expected to reach 740,000 by 2040 [1]. The theranostic of PCa continues to advance rapidly due to more sensitive imaging methods and significant advances in understanding the genomic landscape and the biology of primary and metastatic PCa. These advances are bringing personalized therapies into clinical practice, such as therapies targeting the DNA repair pathway for the treatment of metastatic disease, and combining emerging therapies, such as several novel androgen pathway inhibitors, immunotherapies, and targeted radioisotopes can significantly improve survival in patients with advanced PCa [2]. However, the current clinical indicators cannot meet the needs for prognostication and personalized treatment of PCa patients, and new methods need to be explored to classify patients.

Chromatin and epigenetic disorders mediate tumor progression by generating transcriptional abnormalities and by promoting cancer cell proliferation, and these features have been defined as overall hallmarks of the cancer [3]. Epigenetic modifications include a collection of DNA methylation, chromatin remodels, and so on, but the concern regarding RNA modifications has recently increased [4]. More than 170 RNA modifications have been identified thus far. N^6^-methyladenosine (m6A) has the highest abundance and is well-studied. However, the role of 5-methylcytosine (m5C), another common modification of RNA, remains obscure in many tumors.m5C is a common modification of RNA in all creatures. In eukaryotes, this modification is installed by methyltransferases (writers) such as NSUN/DNMT proteins on diverse RNA species and can be dynamically regulated by demethylases (erasers) such as TET families, and binding proteins (readers) such as YBX1. In humans, m5C methylation is catalyzed by the NSUN protein family. These regulators have different roles; for example, NOP2 (NSUN1) and NSUN5 methylate very conserved residues in 28 s sRNA, whereas NSUN4 and DNA methyltransferase member 2 (DNMT2) target rRNAs and tRNAs [5–8]. In addition, m5C writers have been well-documented in recent years. Recently, some studies have demonstrated that abnormal expression of m5C regulators is associated with various types of cancers, including PCa [9, 10]. Mechanistically, dysfunctional m5C regulator-mediated methylation is correlated with multiple biological processes, including dysregulation of cell death and proliferation, metabolic remodeling, mitochondrial disorders, and even immunomodulatory abnormalities [11, 12]. In recent years, analysis of the whole genome of PCa has indicated that m5C regulatory molecules are new potential susceptibility factors for PCa [13]. However, their association with risk and treatment outcomes in PCa patients is still widely unknown.

In our study, we integrated over 1300 clinical samples with sequencing information, and we studied the profile of m5C regulators from a global multi-omics perspective to assess their overall relevance to PCa clinicopathological characteristics, molecular subtypes, prognosis, androgen receptor signaling inhibitor (ARSI) therapy benefits, and immune phenotypes to determine potential immunotherapy opportunities for PCa.

## Materials and methods

### Data collection and processing

Gene expression sequencing files, DNA mutation, and copy number variation (CNV) data of prostate adenocarcinoma tissues were downloaded free from *The Cancer Genome Atlas* (TCGA, http://portal.gdc.cancer.gov/) project and processed by a series of functions in the R package GDCRNATools [14]. A single-cell RNAseq for different cell types in healthy human prostates was downloaded and integrated from the GenitoUrinary Development Molecular Anatomy Project (GUDMAP) database [15].

Five independent external PCa validation cohorts with detailed biochemical recurrence (BCR) status and survival data in the same sequencing platform, namely, GSE54460, DKFZ, Stockholm, CPC, and SU2C-PCF-2019 were downloaded from the Gene Expression Omnibus (GEO) and cBioPortal (https://www.cbioportal.org/).

Three immunotherapy cohorts from three different cancers were downloaded from the GEO database, including GSE91061 (melanoma), GSE135222 (Non-small cell lung cancer), and Checkmate cohort (advanced renal carcinoma). RNA-seq and RIP-seq of ALYREF were uploaded to GEO (GSE195701).

Detailed information about these cohorts was in Additional file 1: Table S1, and the workflow of this study was shown in Additional file 2: Figure S1.

### Unsupervised clustering pattern of 13 m5C regulators

An unsupervised clustering algorithm was applied to cluster analysis of m5C RNA-modified “writers” consisting of 7 molecules (NOP2, NSUN2, NSUN3, NSUN4, NSUN5, NSUN7, and TRDMT1), 4 m5C erasers (TET1, TET2, TET3, and ALKBH1), 2 readers including YBX1 and ALYREF from previous studies [12, 16]. Unsupervised clustering was applied to detect the robust clustering of prostate adenocarcinoma. We used the "cola" package for the above steps [17], we selected a maximum evaluated k of 6, the mcluster algorithm, and conduct 1000 repetitions for each clustering method to ensure stability and Optimization of the classification.

### Functional enrichment analysis

We identified the differentially expressed genes (DEGs) between m5C clusters and the m5C score groups by using the limma R package. The significance for determining DEGs was set as adjusted P < 0.05 and |logFC|> 1.5. To figure out the differences of RNA m5C modification regulators in biological processes, we used the “GSVA” R package to conduct Gene set variation (GSVA) analysis [18]. The gene sets for the GSVA assay were downloaded from MsigDB. The "ClusterProfiler" R package was used to functionally annotate 13 RNA m5C regulators with Kyoto Encyclopedia of Genes and Genomes (KEGG), gene ontology (GO), and hallmarks analyses [19].

### Construction of m5C score

m5C regulators with FPKM > 1 was analyzed with Cox regression; P < 0.05 was used as the significance cut-off to identify candidates associated with BCR, meanwhile, the median of gene expressions was used to confirm whether the sample belonged to the high-expression or low-expression group. Thirteen m5C regulators were selected to construct a tenfold cross-validation and penalty LASSO regression prognostic prediction model by R package glmnet. GSE54460 was used as a validation cohort. The m5C score was calculated by expression profile data and coefficient of the corresponding m5C regulators with the formula as follows:

$$m5C\;Score={\sum }_{i=1}^{n}\left(\mathrm{Coef}(i)\times {x}_{i}\right)$$

The n is the number of m5C regulators in the prognostic prediction model, Coef(i) represents the coefficient, and X(i) means the relative expression level of m5C regulators identified by LASSO regression.

### Assessment of the prognostic prediction model

Kaplan–Meier method was employed to draw the survival curves and assess the BCR of high and low-risk score groups. Nomogram Construction by “rms” R package.

### GDSC drug sensitivity and TIDE analysis

To further investigate the drug sensitivity correlation of m5C score in PCa, the effects of the m5C score on drug response from the Genomics of Drug Sensitivity in Cancer (GDSC, https://www.cancerrxgene.org/) database. Furthermore, the T-cell dysfunction and exclusion score was used predict the clinical response in treatment of potential immune checkpoint blockade.

### Immune analysis by CIBERSORT and ssGSEA

We used “IOBR” package to calculate the tumor-infiltrating immune cells (TIICs) in the tumor samples [20]. Normalized gene expression profiles and LM22 signature matrix at 1,000 permutations were used to run the CIBERSORT algorithm. The result of running the ssGSEA was conducted to study the difference of microenvironments between the two m5C score groups.

### Cell lines

BPH-1 and 22Rv1 cell lines were recovered from a Liquid nitrogen refrigerated storage tank in the Central Laboratory of Guangzhou First People's Hospital. LNCaP, C4-2, PC-3, and DU145 cell lines were purchased from BeNa Culture Collection. BPH-1, LNCaP, C4-2, and 22Rv1 cells were cultured in RPMI 1640 medium (Meilune, PWL047) and the others (PC-3 and DU145) were cultivated in DMEM (HyClone, SH30022.01). All media were supplemented with 10% fetal bovine serum (FBS, Holocene, HF1054-05) and 1% Penicillin and Streptomycin (Gibco, 15140122). All cell lines were cultured in at 37 °C with 5% CO^2^.

### RNAi transfection and Western blot

The small interfering RNAs (siRNA) targeting ALYREF transcript were synthesized by GenePharm company. LNCaP and C4-2 cells were transfected with siRNAs using siRNA-Mate (GenePharm, G04002). The oligo sequences of all the siRNAs are listed in Additional file 1: Table S2. All transfected cells were lysed by RIPA buffer with PMSF (Boster, AR1192) for total protein extraction. The protein lysates were separated on SDS-PAGE gels and transferred to PVDF membranes (Millipore, FFP39), followed by blocking with 5% skimmed milk and incubated with anti-ALYREF (Abcam, ab6141) and anti-β-actin (Santa Cruz, sc-47778) antibodies at 4 °C overnight. After incubated with secondary antibodies at room temperature for 1 h, the membranes were visualized in a chemiluminescence system.

### In vitro cell function assays

Cell viability was evaluated by CCK-8 kit (Meilune, MA0218) at a sequential time point including 0 h, 24 h, 48 h, 72 h, and 96 h. The plate colony formation assay was used to evaluate the clonogenic ability of the cells. Transwell assay was performed to detect the invasive ability of cells. Each assay was performed in triplicate and the detailed protocols can be referred to in our previous study [21].

### In vivo experiments

All animal experiments were approved by the Animal Care Committee of Guangzhou First People's Hospital. Ten four-week male BALB/c nude mice were purchased from Guangdong Medical Laboratory Animal Center. 3 × 10^6^ C4-2 cells mixed with Matrigel (Corning, 354,277) was injected subcutaneously into the backside of the nude mice. Then the mice were randomized into two groups, followed by treated with si-NC or si-ALYREF#615 mixed with RNAi-Mate (GenePharm, G04001) in situ injection for three weeks. Tumor volume was inspected and calculated every 4 days. Finally, the mice were sacrificed and the tumor was weighed.

### Immunohistochemistry

Immunohistochemistry (IHC) was conducted to investigate the expression level of ALYREF in Prostate cancer Tissue microarrays (TMA, PR808b; Alenabio Biotech, Xi’an, China) according to the protocol described previously, and the procedures were approved by the Ethics Committee of Guangzhou First People's Hospital [22].

### RNA immunoprecipitation, RNA extraction, library preparation, and sequencing

RNA immunoprecipitation (RIP) was performed on the C4-2 cell line. The cell was treated with cell lysis buffer. The 10% lysis sample was stored and named “Input”, and 90% was used in immunoprecipitation reactions with anti-ALYREF antibody) and named “IP”, and 10% was incubated with rabbit IgG (Cell Signaling Technology) as a negative control and named “IgG”, respectively. More details were shown in our previous study [21].

Total RNAs were extracted from the C4-2 cell line using TRIzol Reagent. DNA digestion was carried out after RNA extraction by DNaseI. RNA quality was determined by with Nanodrop™ One/Onec Spectrophotometer (Thermo Fisher Scientific Inc). Qualified RNAs were finally quantified by Qubit3.0 (QubitTM RNA Broad Range Assay kit, Life Technologies.) 2 μg total RNAs were used for stranded RNA sequencing library preparation (KCTM Stranded mRNA Library Prep Kit for Illumina^®^, NO. DR08402, Wuhan Seqhealth Co., Ltd. China) following the instruction. PCR products corresponding to 200–500 bps were enriched, quantified, and finally sequenced on Novaseq 6000 sequencer (Illumina) with PE150 model.

### Statistical analysis

R (version 4.1.2) and related packages were applied to all statistical analyses. We used the t-test to compare continuous variables between two groups if they fit the normal distribution, and the Wilcoxon test was used to compare two independent non-parametric samples. The Pearson coefficients were used to analyze the correlation between continuous variables. For the m5C score, we applied the “survcutpoint” function to determine the cutoffs for low- and high- m5C score groups, except in the TCGA-PRAD cohort, in which we defined the median as the cutoff. And the Kaplan-Meier survival curves were verified with the log-rank test. We used chi-square to analyze the difference between categorical variables and mean ± standard deviation was used to describe the continuous variables in normal distribution while the median (range) was applied to testify the continuous variables in abnormal distribution. P < 0.05 was regarded as statistically significant.

## Results

### The landscape of m5C regulators in prostate adenocarcinoma

**Fig. 1:**
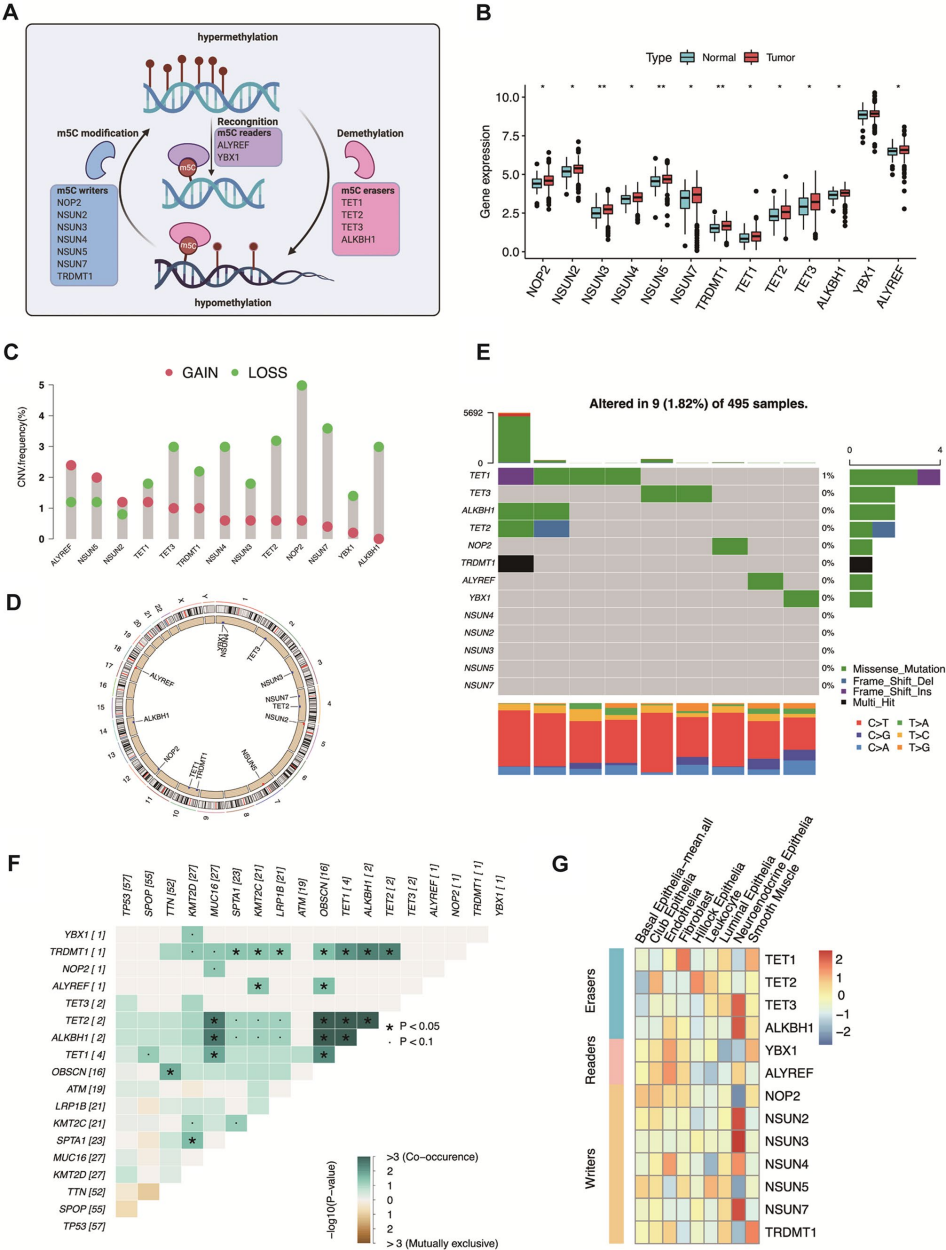
The expression and alteration landscape of m5C regulators in PCa. **A** Diagram of m5C modification regulators and their biological functions. **B** The mRNA expression of m5C regulators between PCa tissues and benign prostatic tissues in the TCGA database, the asterisks represented the statistical P-value (*P < 0.05; **P < 0.01; ***P < 0.001). **C** The diagram exhibited the CNV mutation frequency of 13 m5C regulators. The column length represented the alteration frequency. The green dot denotes the deletion (LOSS) frequency; the pink dot stands for amplification (GAIN) frequency. **D** The CNV mutation sites of m5C regulators on chromosomes. **E** 9 of 495 (1.82%) PCa patients experienced genetic alterations of the 13 m5C regulators, most of which included missense mutations. The percentages on the right side indicated the mutation frequencies of m5C regulators, respectively. Each column represented an individual patient. **F** The mutually exclusive or co-occurrence relationship between the m5C regulators and top mutant genes in PCa, ·P < 0.1; *P < 0.05. **G** The heatmap shows the single-cell sequencing expression profile of 13 m5C regulators in different cell types in prostatic tissues

A total of 13 m5C regulators, including 7 writers, 4 readers, and 2 erasers from published articles were included in our study (Fig. 1A) [12, 16]. To summarize the role of m5C regulators in PCa, we first investigated their mRNA expression. Compared with benign prostate tissues, PCa tissues had remarkably higher expression of 7 m5C writers (NOP2, NSUN2, NSUN3, NSUN4, NSUN5, TRDMT1, and NSUN7), 4 m5C erasers (TET1, TET2, TET3, ALKBH1), and 2 readers (ALYREF, YBX1) (Fig. 1B). As shown in Fig. 1C and 1D, the 13 m5C regulators exhibited a different CNV frequency, and ALYREF had the highest gain of CNV frequency. From the 495 PCa samples in The Cancer Genome Atlas (TCGA) cohort, only 9 showed mutations of m5C regulators, resulting in a low frequency of approximately 1.82% (Fig. 1E). Somatic interaction analysis found that mutations in m5C regulators cooccur with the most common molecular mutations in PCa (Fig. 1F).

We further analyzed the expression level of m5C regulatory molecules in different molecular subtypes of PCa. As shown in Additional file 2: Fig. S2, Ten of the 13 molecules (not NOP2, NSUN5, and ALKBH1) were significantly overexpressed in ERG fusion subtypes. ALYREF was also significantly overexpressed in TP53-mutant and patients with microsatellite instability (MSI) (Additional file 2: Fig. S2A-C). Nonetheless, survival analysis showed that high expression levels of five m5C regulators indicated poor BCR free survival, and the other five regulators seemed to be protective factors (Additional file 2: Fig. S2D and E).

In addition, we demonstrated the overexpression of m5C regulators with single-cell RNA (scRNA) sequencing of prostate cells with the uniform manifold approximation and projection (UMAP) and t-distributed stochastic neighbor embedding (t-SNE) algorithms and classified the cells into nine different cell types, including neuroendocrine epithelial cells, luminal epithelial cells, and fibroblasts (Additional file 2: Fig. S3; Additional file 1: Table S3). In the single cell sequencing, m5C regulators, including ALYREF, TET3, NUSN2, and NUSN3, were expressed in different cell types (Fig. 1G). In summary, m5C regulators may be potential prognostic and therapeutic predictors in PCa.

### m5C clusters and the characteristics mediated by the 13 m5C regulators

**Fig. 2:**
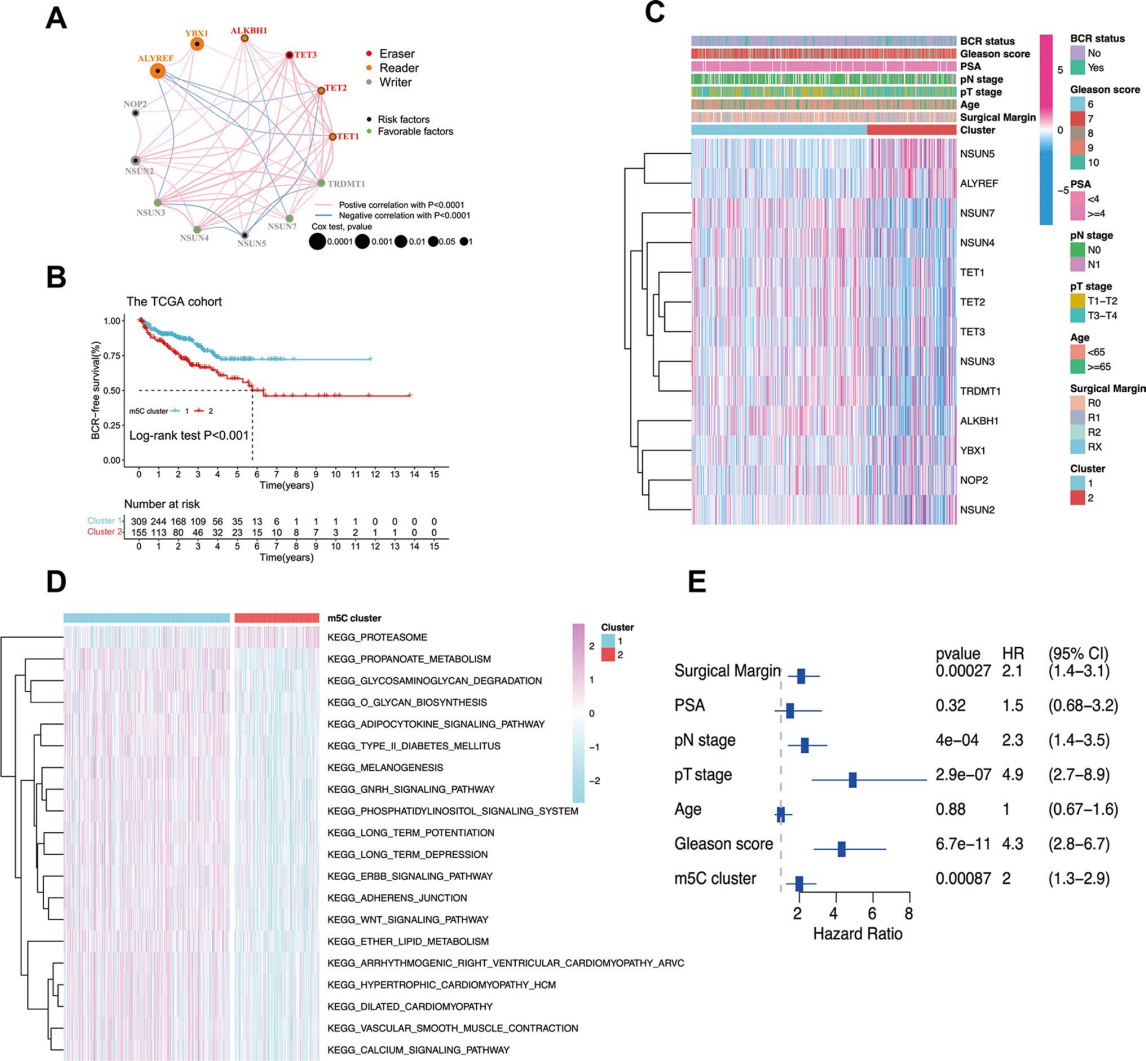
The clinical relevance of the m5C cluster in the TCGA-PRAD. **A** The interaction network among m5C regulators and their individual prognostic value. The circle size indicates the p-value of the Cox test, and the lines in the network indicate their interactions. Pink lines show a positive correlation, while blue lines represent a negative correlation; purple dots indicate risk factors, while green dots represent favorable factors. **B** The survival analysis of the two m5C clusters. **C** The heatmap shows the association between the 13 m5C regulators’ expression profile and clinical traits plus the m5C cluster. **D** The heatmap shows the GSVA score profile of representative pathways in 2 m5C clusters. **E** The forest plot integrates the univariable Cox analysis results of the m5C cluster and clinical characteristics

The TCGA-PRAD with BCR survival information was employed in our study. Univariate Cox regression model and network analyses uncovered the landscape of m5C regulators and their interactions and the prognostic significance of these factors in PCa patients (Fig. 2A; Additional file 1: Table S4). Our study revealed that ALYREF was the most significant risk factor for PCa patients, as shown in Additional file 2: Fig. S2D and E. In order to investigate the role of 13 m5C regulators in prostate cancer, we attempted to cluster these samples based on their expression levels using an unsupervised clustering approach. As demonstrated in Additional file 2: Fig. S4A-F, the TCGA-PRAD samples were classified into different clusters. Interestingly, when the PCa samples were divided into two distinct clusters by the expression levels of the 13 m5C regulators, the clustering was superior and could predict the prognosis of PCa patients. Thus, 309 PCa patients were divided into m5C cluster 1, and the other 155 patients were divided into m5C cluster 2. As shown in Fig. 2B, PCa patients in m5C cluster 2 were more prone to BCR than those in m5C cluster 1. As shown in Fig. 2C, the mRNA expression levels of the 13 m5C regulators were obviously different between the two m5C clusters. To investigate the biological behaviors among the two distinct m5C clusters, we performed gene set variation analysis (GSVA), gene ontology (GO), and Kyoto Encyclopedia of Genes and Genomes (KEGG) enrichment analyses, as shown in Fig. 2E and Additional file 2: Fig. S5B-C, and the clinical relevance of the m5C clusters is shown in Additional file 2: Fig. S5A. Notably, patients in m5C cluster 2 with poor outcomes showed enrichment of activation of the KEGG proteasome pathway (Fig. 2D; Additional file 1: Table S5), which is closely related to PCa castration resistance [23]. Further univariate Cox regression analysis indicated that the m5C clusters were significant independent prognostic predictors (Fig. 2E).

### m5C signature development, validation, and functional enrichment assessment

Patients without BCR follow-up information were excluded. In total, 464 PCa patients from the TCGA-PRAD and 396 PCa patients from the GSE54460, Stockholm, CPC, and DKFZ cohorts were included as the training cohort and the validation cohort, respectively. We applied LASSO regression to avoid overfitting of the m5C score model (Additional file 2: Fig. S6A and B), resulting in seven candidate prognostic genes: NOP2, NSUN4, TRDMT1, TET1, TET3, ALKBH1, and ALYREF (Additional file 1: Table S6). The landscape of the 13 m5C regulators between the m5C score groups and the relationships between the m5C clusters and the m5C score are depicted in Additional file 2: Fig. S7A-C. The results of GSVA comparing the m5C score groups of the TCGA cohort are shown in Additional file 2: Fig. S7D. Multiple metabolic pathways were differentially activated between the m5C score groups, such as fatty acid metabolism and pyruvate metabolism.

**Fig. 3:**
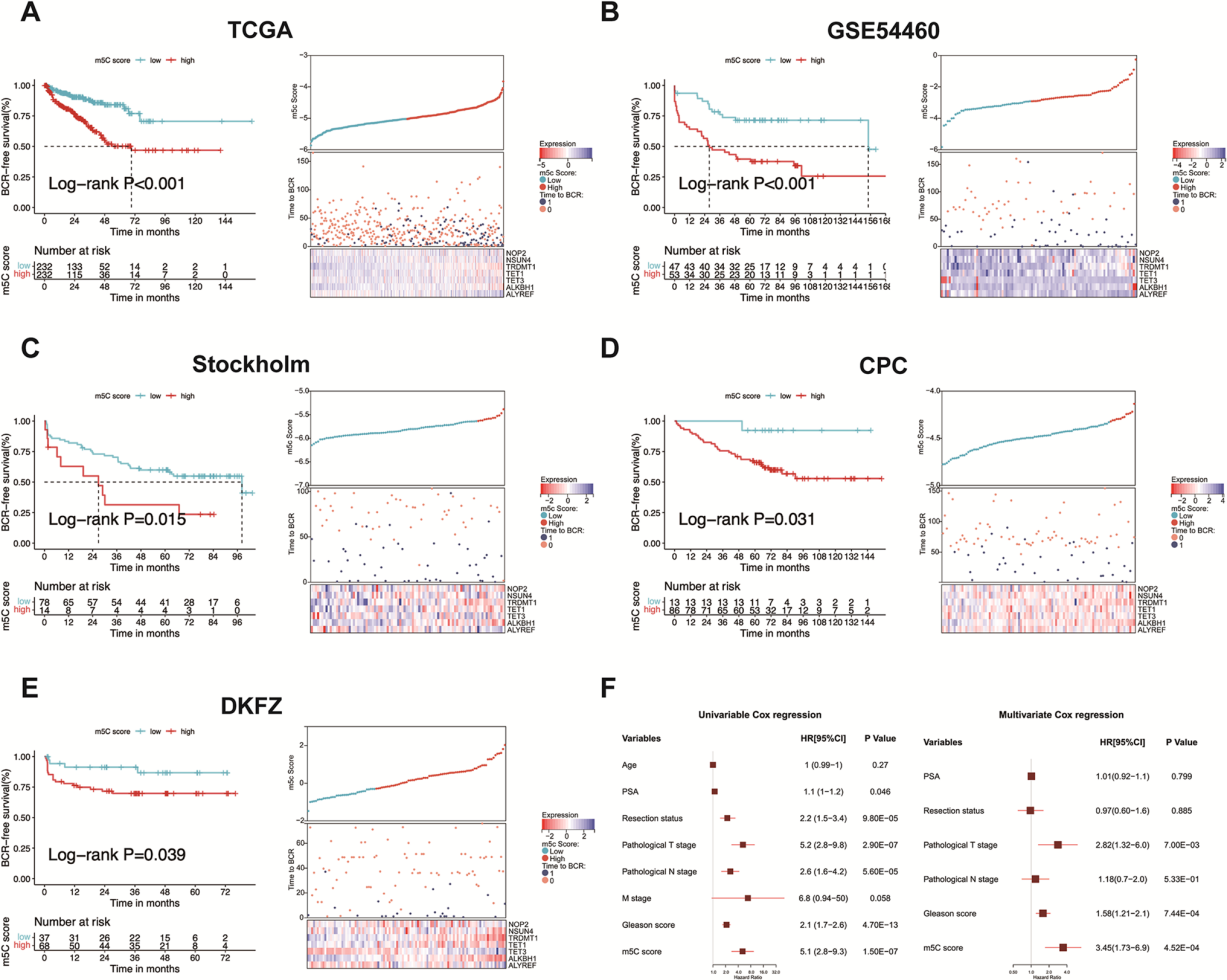
The performance of the m5C score signature in predicting the BCR free survival of PCa. **A** The Kaplan Meier survival analysis of the m5C score and the expression heatmap of seven genes in the signature using the TCGA-PRAD cohort. (P < 0.001, Log-rank test). **B** The Kaplan Meier survival analysis of the m5C score and the expression heatmap of seven genes in the signature using the GSE54460 cohort (P < 0.001, Log-rank test). **C** The Kaplan Meier survival analysis of the m5C score and the expression heatmap of seven genes in the signature using the Stockholm cohort (P = 0.015, Log-rank test). **D** The Kaplan Meier survival analysis of the m5C score and the expression heatmap of seven genes in the signature using the CPC cohort (P = 0.031, Log-rank test). **E** The Kaplan Meier survival analysis of the m5C score and the expression heatmap of seven genes in the signature using the DKFZ cohort (P = 0.039, Log-rank test). **F** The forest plots show the univariable and multivariable Cox analysis results for the m5C score and clinical traits

To develop the m5C signature score model, 4 other external cohorts from open-access databases were included as the validation cohorts. Patients were divided into two different groups according to the m5C signature score. In both the TCGA and validation cohorts, patients in the high m5C score group exhibited poorer BCR free survival than those in the low m5C score group (Fig. 3A-E). Univariate and multivariate Cox regression analyses of the TCGA cohort suggested that the m5C score was an independent prognostic factor after adjusting for PSA level, age, surgical resection margin status, Gleason score, and TNM stage (Fig. 3F). The nomogram generated through multivariate Cox analysis and the calibration plot are shown in Additional file 2: Fig. S6C.

We compared the enriched GO and KEGG pathways and the mutation profiles between the two m5C signature score groups. In particular, immune-related KEGG pathway terms PD-L1 expression and PD-1 checkpoint pathway in cancer were significantly differentially enriched between the two m5C score groups (Additional file 2: Fig. S8A and B; Additional file 1: Table S7). Moreover, patients in the high m5C score group had a higher TP53 mutation rate (12% versus 8%, Additional file 2: Fig. S8C and D). Collectively, the m5C score based on m5C regulators reflected the clinical and biological characteristics of PCa patients.

### Role of the m5C signature in predicting endocrine therapy and chemotherapy benefits in PCa

**Fig. 4:**
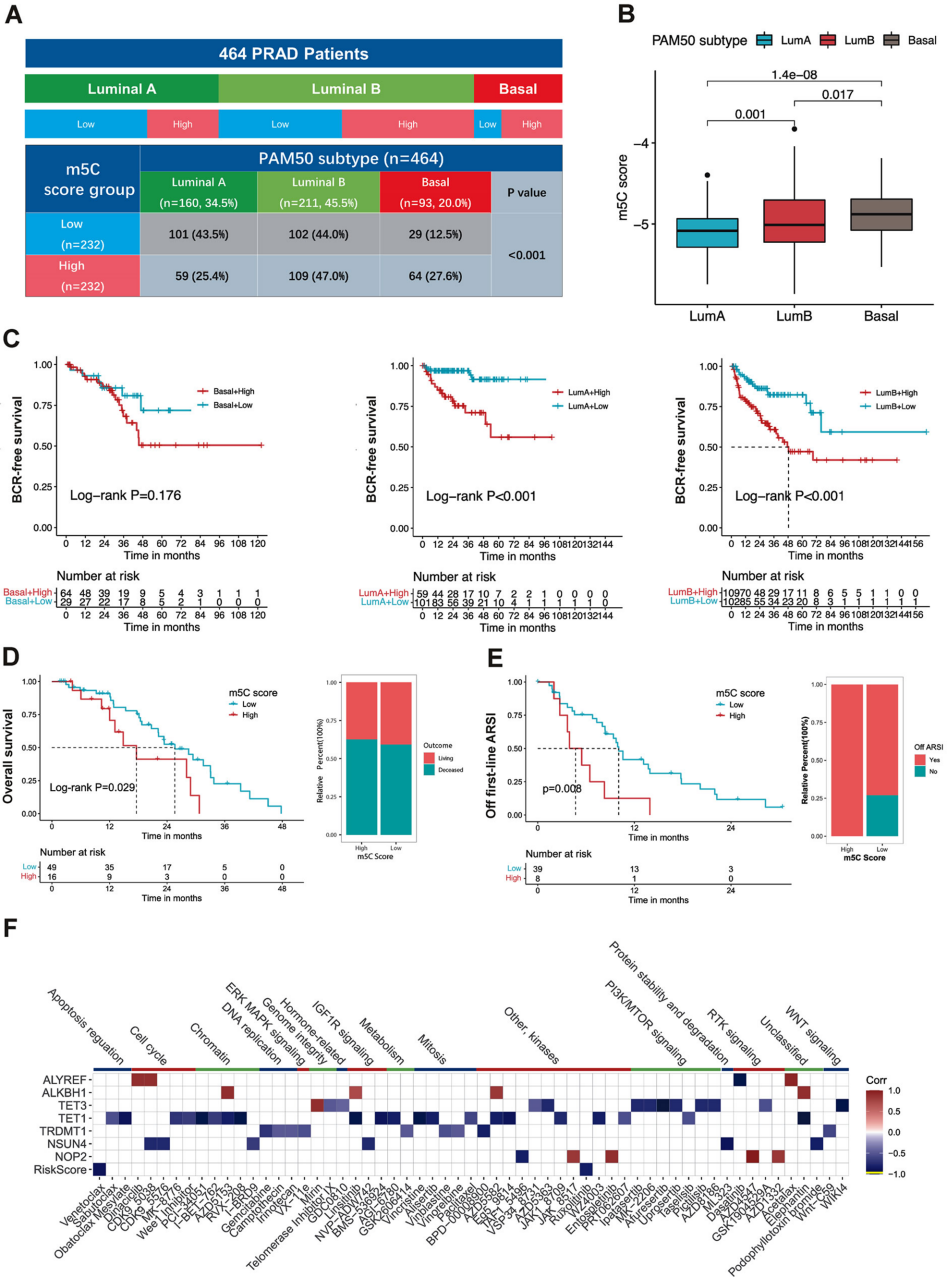
The m5C score correlated with PAM50 subtypes, prognosis, and the response to ARSI therapy. **A** The correlation between the m5C score groups and PAM50 subtypes in TCGA-PRAD cohort (P < 0.001, Chi-square test). **B** The differences in the m5C score among three PCa PAM50 subtypes. **C** The diagrams display the Kaplan–Meier survival analyses (BCR free survival) for PCa patients of two m5C score subgroups within each PAM50 subtype. The numbers of patients in the basal, luminal A, and luminal B subtypes are 93, 160, and 211, respectively. The high m5C score group showed a significantly worse prognosis in both luminal A and luminal B subtypes (P < 0.001, Log-rank test). **D** The Kaplan–Meier survival analysis (P = 0.029, Log-rank test) analysis, and outcome constitution (living or deceased) of PCa patients treated with first-line ARSI in the two m5C score subgroups. **E** The Kaplan–Meier curve (P = 0.008, Log-rank test), and the ARSI-resistance (off ARSI) constitution of PCa patients in the two m5C score subgroups. **F** The heatmap shows the correlation between the m5C score signature and drugs’ IC50 based on the GDSC database in PCa cell lines (colored squares indicate statistically significant correlations with P < 0.05). The squares with gradients of red indicate positive correlations to different extents. The squares with gradients of blue indicate negative correlations to different extents

The PAM50 classification scheme in malignancies has been shown to be biologically important in determining prognosis and therapy response [24], and the PAM50 molecular subtypes of PCa mainly include the luminal A (LumA), luminal B (LumB), and basal phenotypes. In our study, we found that the m5C score was related to the PAM50 molecular subtypes of PCa patients. As shown in Fig. 4A, we found that luminal subtypes (LumA, n = 160, 34.5%; LumB, n = 211, 45.5%) were predominant in the 464 TCGA PCa patients with BCR prognostic follow-up data. According to the results, the m5C scores were significantly higher in basal and LumB patients than in LumA patients (Fig. 4B). We found that the high m5C score group in the three PAM50 subtypes consistently had poor outcomes (Fig. 4C).

ARSI is one of the first-line nonsurgical treatments for PCa patients, and the application of new ARSIs will undoubtedly greatly improve the treatment of PCa. Therefore, we investigated the value of the m5C score in predicting patient response to ARSI in an independent ARSI cohort [25]. Notably, in the ARSI cohort, patients who received ARSI treatment with low m5C scores were likely to have better overall survival (Fig. 4D). Moreover, patients with high m5C scores exhibited poor benefit from ARSI treatment and more easily developed drug resistance (Fig. 4E). Molecular factors such as cell cycle progression (CCP) scores, RB-loss scores have been reported to play a role in ARSI treatment in PCa [25]. Consistent with this, the CCP and RB1-loss scores were positively correlated with the m5C score (Additional file 2: Fig. S9A-D).

Finally, the correlation between the m5C score and drug sensitivity was revealed (Additional file 1: Table S8). Notably, the m5C score was negatively correlated with resistance to ruxolitinib and venetoclax in six PCa cell lines (Fig. 4F, P < 0.05). Taken together, the results suggested that the m5C score is associated with the response to ARSI and other therapies.

### Association of the m5C signature with PCa immune phenotypes and the response to PD1/PD-L1 inhibitors

**Fig. 5:**
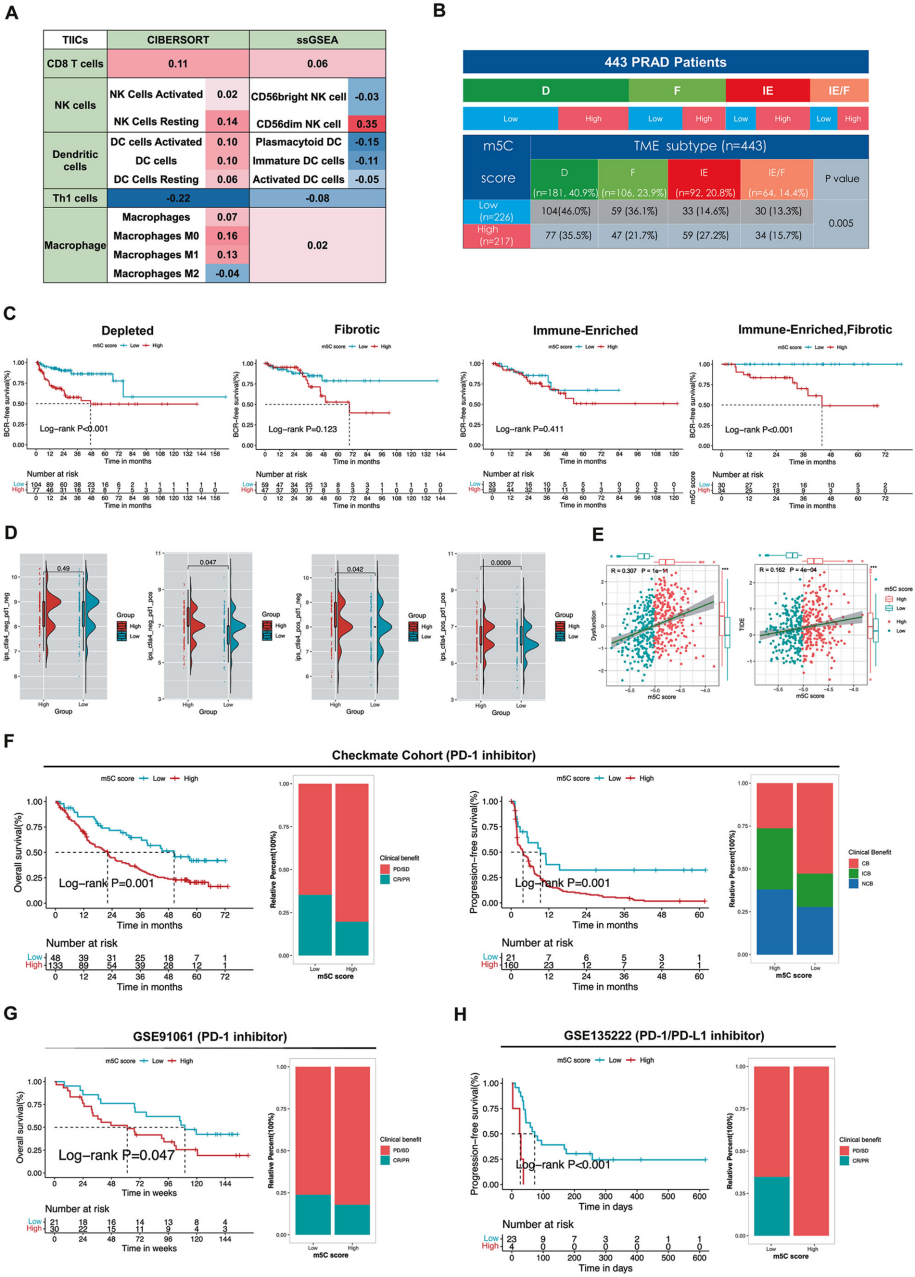
The m5C score correlated with immune phenotypes, prognosis, and the response to immunotherapy. **A** The correlation between the m5C score and the infiltration levels of five immune infiltration cells which were calculated using ssGSEA and cibersort algorithms. **B** The correlation between the m5C score subgroups and immune phenotypes (TME subtypes) in the TCGA-PRAD cohort (P = 0.005, Chi-square test). **C** The diagrams display the Kaplan–Meier survival analyses (BCR free survival) for PCa patients of two m5C score subgroups within each TME subtype, Log-rank test (Depleted, P < 0.001; Fibrotic, P = 0.123; Immune-Enriched, P = 0.411; Immune-enriched with Fibrotic, P < 0.001). **D** The respective correlations between the m5C score and the TIDE and dysfunction scores in the TCGA-PRAD cohort. R represents Pearson’s correlation coefficient, and P stands for P-value. *P < 0.05, **P < 0.01, ***P < 0.001. **E** The differential expression of PD-(L)1 between the m5C score subgroups in three PD-(L)1 inhibitor treatment cohorts (Checkmate cohort, advanced renal cell carcinoma; GSE91061, non-small cell lung cancer; GSE135222, myeloma. ns, P ≥ 0.05; *P < 0.05; **P < 0.01; ***P < 0.001). **F** The Kaplan–Meier curves (P = 0.001, Log-rank test) and the clinical benefit proportions of patients treated with Nivolumab of the two m5C score subgroups in the Checkmate cohort. **G** The Kaplan–Meier curves (P = 0.047, Log-rank test) and the clinical benefit proportions of patients treated with the PD-1 inhibitor of the two m5C score subgroups in the GSE91061 cohort. **H** The Kaplan–Meier curves (P = 0.047, Log-rank test) and the clinical benefit proportions of patients treated with the PD-(L)1 inhibitor of the two m5C score subgroups in the GSE135222 cohort

Immunotherapy has proven to be of vital importance in various malignancies. To explore the different effects of the m5C signature on the tumor microenvironment (TME), we used single-sample gene set enrichment analysis (ssGSEA) and the CIBERSORT algorithm to compare immune cell infiltration between the low and high m5C score groups (Additional file 1: Table S9). As shown in Additional file 2: Fig. S10A, PCa patients with high m5C scores mainly had a tumor-suppressor microenvironment, as myeloid-derived suppressor cells (MDSCs) were the major infiltrating cell. However, to our surprise, the high m5C score group also showed an increased proportion of CD56dim natural killer (NK) cells, which have an antitumor function (Fig. 5A; Additional file 2: Fig. S11A-D). These results suggest that the immune microenvironment of PCa patients is complicated, but further evidence is required.

A previous study showed that conserved TME subtypes correlate with cancer progression [26]. Therefore, we assessed the TME subtypes of PCa to explore whether the m5C score has an impact on prognosis. As shown in Fig. 5B, we found that of four TME subtypes (depleted (D), immune-enriched without fibrotic (IE), fibrotic (F), and immune-enriched with fibrotic (IE/F)), the depleted subtype (representing cold tumors), was the most common in localized PCa. In addition, our results demonstrated that the infiltration of immune cells was more abundant in the high m5C score group, and the m5C scores of the IE subtype were higher than those of the other three subtypes (Additional file 2: Fig. S11A). PCa patients with the IE subtype and low m5C scores did not show a survival advantage (Fig. 5C). Moreover, immune activation-related genes, immune checkpoint-related genes, and macrophage polarization-related genes were upregulated in PCa patients with high m5C scores (Additional file 2: Fig. S11B-D). Taken together, these results suggest that PCa patients with high m5C scores are more likely to have an immune-enriched microenvironment and have a worse prognosis than those with low m5C scores.

Prostate cancer is commonly classified as a "cold" tumor; however, based on our immune analysis as mentioned above, we found that some samples still exhibit immune cell infiltration. Nonetheless, there is a paucity of follow-up data on immunotherapy for prostate cancer in public databases. Therefore, we utilized publicly available data from other cancers to explore the association between m5C score and the prognosis of immunotherapy as a preliminary step. To more comprehensively reveal the correlation between the m5C score and immunotherapy response, the T cell dysfunction and exclusion (TIDE) score was utilized to predict the response to immune checkpoint blockade (ICB) in the different m5C score groups of patients with PCa [27]. Notably, the high m5C score group had higher TIDE scores, indicating poor response to ICB, suggesting that a high m5C score may predict worse outcome (Fig. 5D). Moreover, we took advantage of three PD-1 and/or PD-L1 intervention cohorts, an advanced renal cell carcinoma (RCC) Checkmate cohort, GSE91061 (non-small-cell lung cancer (NSCLC), and GSE135222 (myeloma), to determine whether the m5C score could predict the therapy response in different solid cancers. PD-(L)1 mRNA was overexpressed in the high m5C score groups of the three immunotherapy validation cohorts (Fig. 5E). Patients with low m5C scores showed significantly better overall and progression-free survival as well as clinical benefit (Fig. 5F-H). These results have significance for clinical practice and suggest that further research is needed to confirm the importance of the relationship between the m5C signature and ICB.

### Validation of the therapeutic and prognostic value of ALYREF in PCa

In previous results, we utilized several in silico methods to corroborate the role of m5C in prostate cancer. To further enhance the reliability of our results, we endeavored to validate the hub gene that was identified through our analysis. As presented in Fig. 1B, ALYREF exhibited the highest expression level among the differentially expressed genes. Moreover, ALYREF ranked second in coefficient among the m5C-related genes, just slightly below the top-ranked gene (Additional file 1: Table S6). Given these findings, we selected ALYREF as the hub gene for further investigation.

**Fig. 6:**
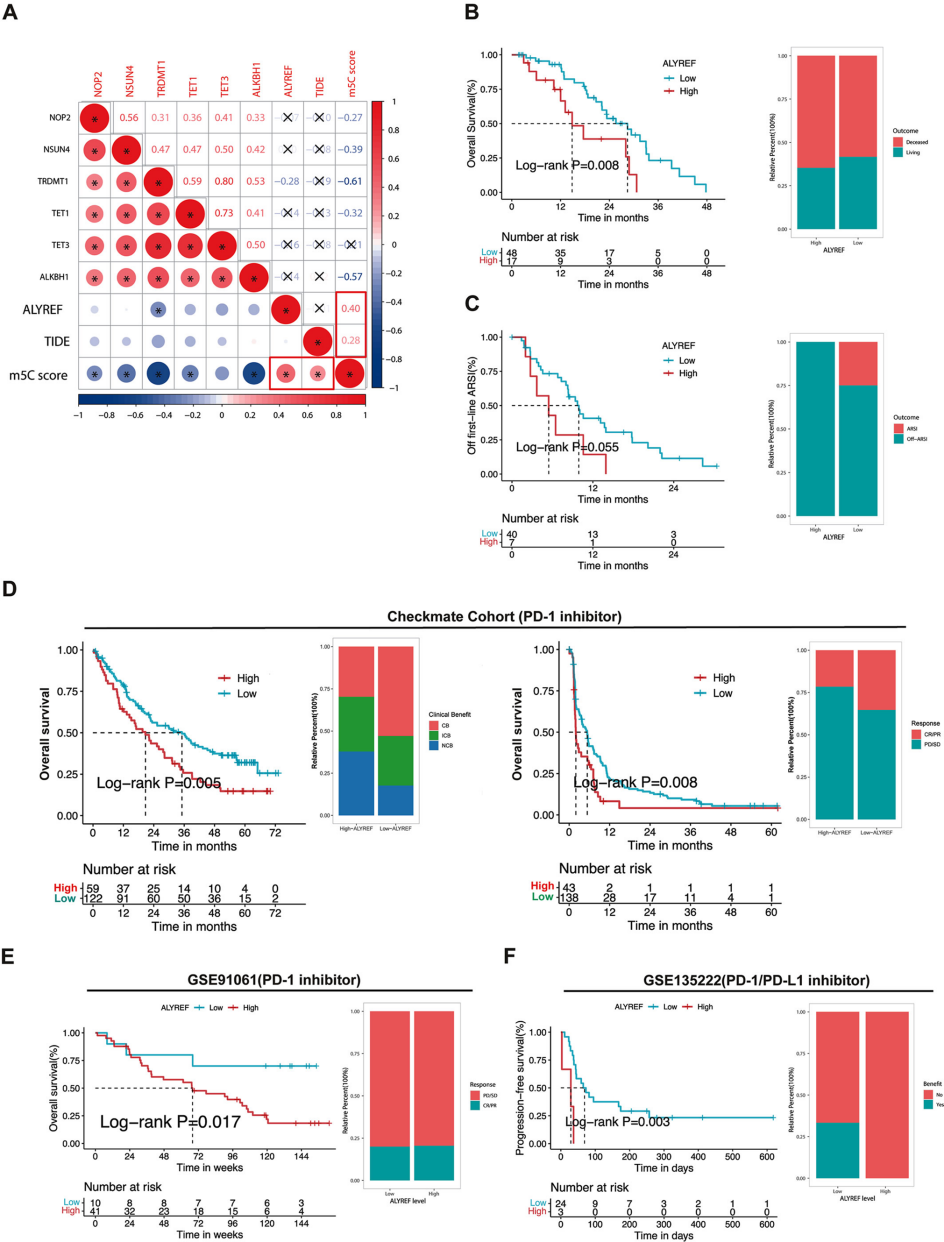
The prognosis and therapeutic opportunity relevance of the m5C reader ALYREF expression level. **A** The correlation heatmap revealed ALYREF was indirectly positively correlated with TIDE in PCa patients, whose TIDE is larger than 1. The upper zone shows the Pearson correlation coefficient with P < 0.05. **B** The Kaplan–Meier curves (P = 0.008, Log-rank test) and the clinical outcome (living or deceased) proportion of PCa patients treated with first-line ARSI of the two ALYREF subgroups. **C** The Kaplan–Meier curves (P = 0.055, Log-rank test) and the ARSI-resistance (off ARSI) proportion of PCa patients treated with first-line ARSI of the two ALYREF subgroups. **D** The Kaplan–Meier curves (P = 0.005 in OS and P = 0.008 in PFS, Log-rank test) and the clinical benefit proportion of patients treated with Nivolumab of the two ALYREF subgroups in the Checkmate cohort. **E** The Kaplan–Meier curves (P = 0.017, Log-rank test) and the clinical benefit proportion of patients treated with the PD-1 inhibitor of the two ALYREF subgroups in the GSE91061 cohort. **F** The Kaplan–Meier curves (P = 0.003, Log-rank test) and the clinical benefit proportion of patients treated with the PD-(L)1 inhibitor of the two ALYREF subgroups in the GSE135222 cohort

In Fig. 6A, we observed that only ALYREF displayed a positive correlation with both the m5C score and TIDE score. High mRNA expression of ALYREF also predicted poor prognosis and lack of clinical benefit in patients with ARSI treatment (Fig. 6B and C). Moreover, in the three immunotherapy validation cohorts, high expression of ALYREF strongly indicated poor prognosis and lack of clinical benefits in patients treated with PD-1/PD-L1 inhibitors. In addition, pancancer progression-free interval (PFI) analysis in TCGA revealed that ALYREF is an unfavorable factor in kidney chromophobe, uveal melanoma, glioma, and hepatocellular carcinoma (Additional file 2: Fig. S12E). Our results suggest that ALYREF could be a potential prognostic and therapeutic predictor in PCa.

### ALYREF promotes PCa progression in *vitro* and in *vivo*

**Fig. 7:**
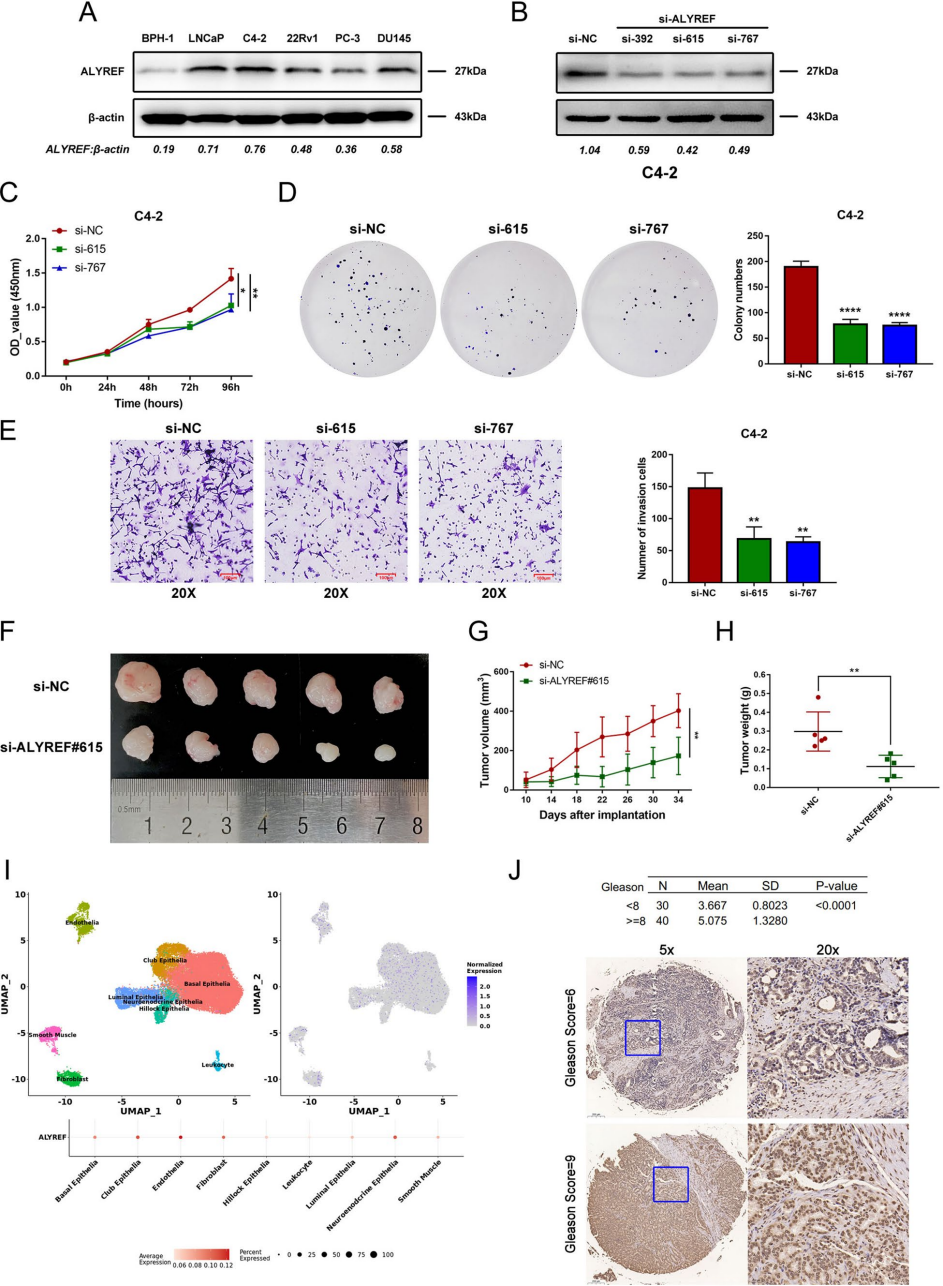
ALYREF promotes prostate cancer progression. **A** Western blotting checked the basal protein levels of ALYREF in the benign prostate epithelial cell line and different PCa cell lines. β-actin was the loading control. **B** Western blotting validated the knockdown efficacy of siRNAs (si-392, si-615, si-767) targeting ALYREF in the C4-2 cell line. β-actin was the loading control. **C** 4-day CCK-8 assay reflected the knockdown of ALYREF attenuated C4-2 cell proliferation. **D** Plate colony formation assay showed silencing ALYREF downregulated C4-2 cell colony formation ability. **E** Transwell assay confirmed silencing ALYREF diminished C4-2 cell invasion ability. **F** In *vivo* experiments showed that downregulation of ALYREF inhibited the subcutaneous xenografts’ growth with enzalutamide. **G** Subcutaneous xenografts on si-ALYREF#615 mice were smaller than that on si-NC mice in terms of volume. **H** Subcutaneous xenografts on si-ALYREF#615 mice were lighter than that on si-NC mice in terms of weight. **I** Single-cell sequencing analysis exhibited the average expression of ALYREF in each type of cells (9 kinds). **J** IHC assay confirmed that the expression of ALYREF positively relates to the Gleason score. Error bar indicates mean ± SD. *P < 0.05, **P < 0.01

To determine the potential role of ALYREF in PCa in *vitro* and in *vivo*, we first detected its protein expression level in five prostate cell lines, and the results suggested that ALYREF is more overexpressed in C4-2 and LNCaP cells than in BPH-1 cells (Fig. 7A). Therefore, we used three siRNAs to generate loss-of-function C4-2 and LNCaP cell models. Subsequently, si-615 and si-767 were used to effectively knock down the protein expression level of ALYREF (Fig. 7B; Additional file 2: Fig. S12A). CCK-8 and colony formation assays demonstrated that ALYREF inhibition significantly blocked the proliferative abilities of C4-2 and LNCaP cells (Fig. 7C and D; Additional file 2: Fig. S12C and D). Moreover, ALYREF knockdown impaired the migration ability of C4-2 and LNCaP cells (Fig. 7E; Additional file 2: Fig. S12B). In *vivo*, we found that the ALYREF inhibitor si-615 significantly suppressed the growth rate and size of subcutaneous tumors in nude mice (Fig. 7F-H) with enzalutamide. Moreover, UMAP analyses found that ALYREF was mainly expressed in club epithelial cells, endothelial cells, and fibroblasts, and perhaps most importantly, it was highly expressed in neuroendocrine cells (Fig. 7I). Finally, immunohistochemistry (IHC) staining revealed that ALYREF was highly expressed in the high Gleason score group (Gleason score 7 vs. 9) and was mainly located in the nucleus (Fig. 7J; Additional file 1: Table S10).

### Functional enrichment analysis of ALYREF by RNA-seq and RIP-seq

**Fig. 8:**
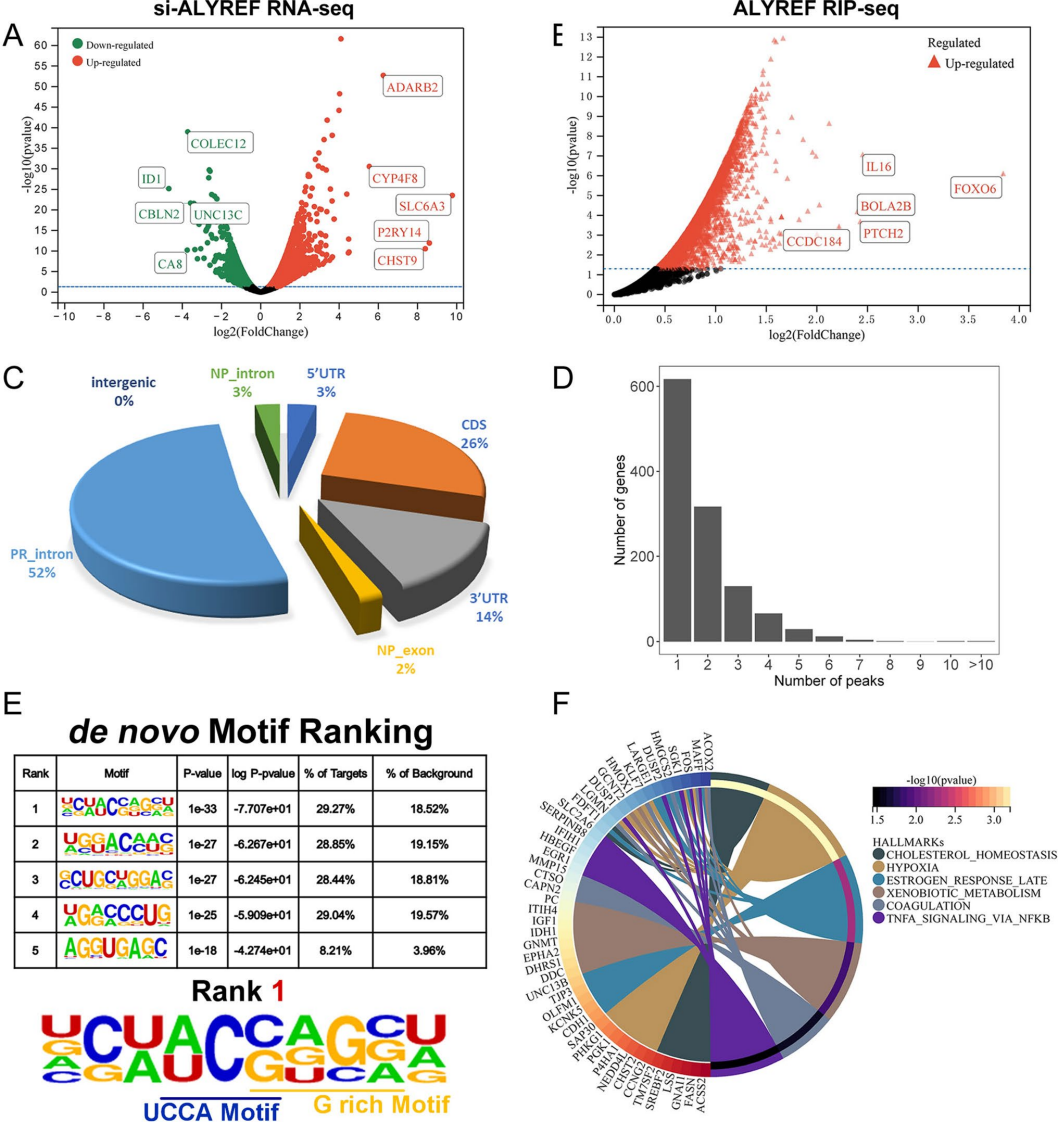
Functional Enrichment Analysis of ALYREF. **A** The volcano plot displayed the differentially expressed genes in the si-ALYREF RNA-seq (fold change > 1, p-value < 0.05). **B** The volcano plot displayed the binding genes in the ALYREF RIP-seq (p-value < 0.05). **C** The pie chart showed the constitution of ALYREF RIP-seq data. **D** The histogram displayed the proportion of genes with different numbers of peaks according to the ALYREF RIP-seq analysis. **E** The top 5 de novo Motif complexes, obtained from the RIP-seq data, were shown. **F** The top 6 hallmarks in the functional enrichment analysis of the RNA-seq and RIP-seq co-analysis

Subsequently, we performed an si-ALYREF RNA-sequencing (RNA-seq) analysis and an ALYREF RNA immunoprecipitation sequencing (RIP-seq) analysis of C4-2 cells. For both analyses, we identified those binding molecules with fold changes exceeding 2 and P < 0.05 as differentially expressed genes (DEGs) (Additional file 1: Tables S11 and 12). The differentially expressed genes derived from si-ALYREF RNA-seq and ALYREF RIP-seq are shown in volcano plots **(**Fig. 8A and B**).** Next, we scrutinized the areas of the RNA sequences that bound to ALYREF in RIP-seq analysis: the PR_intron area accounted for 52% of binding, ranking first, and the NP_exon area accounted for 2%, ranking last (Fig. 8C). In parallel, we examined the binding sites of the selected genes. As shown in the histogram (Fig. 8D), more than 600 genes exhibited only one peak, indicating only one binding site for the ALYREF antibody. Subsequently, we investigated the motifs in these RNA sequences, and we listed the top 5 de novo motif complexes in Fig. 8E. We noticed two m5C-related motifs (the UCCA motif and G-rich motif, which are reportedly related to m5C) in the rank 1 motif complex, as expected. Ultimately, the si-ALYREF RNA-seq and ALYREF RIP-seq analyses identified 241 binding DEGs (Additional file 1: Fig. S13A). Thus, we attempted to explore the molecular roles and mechanisms of these DEGs. We performed three functional enrichment analyses to identify the enriched hallmarks, GO terms, and KEGG pathways of the 241 molecules. The significantly enriched hallmarks (p < 0.05) of these genes included *cholesterol homeostasis*, *late estrogen response*, *hypoxia, UV response*, *TNFA signaling *via* NF-Κb, xenobiotic metabolism and coagulation* (Fig. 8F). In addition, the complete results of the functional enrichment analysis are shown in Additional file 2: Fig. S13B and C; Additional file 1: Table S13.

## Discussion

It was not until 2015 that "epitranscriptome"-related studies began to emerge and had a significant impact on the study of human diseases such as cancers [12, 28]. Increasing evidence has demonstrated that m5C regulators are correlated with prognosis in several cancers [13, 29–32]. Functionally, abnormal expression of m5C regulatory molecules is associated with tumor progression. In our study, we focused on the m5C regulator signature and the landscape of m5C modification patterns in PCa molecular subtypes and identified the overall clinical value of a m5C regulator signature to enhance management of disease progression, therapeutic response, and outcomes in PCa patients.

PCa is ranked 5^th^ in cancer-related mortality in men worldwide [33]. Although the prognosis of PCa has improved over recent decades, most patients inevitably develop castration resistance. New gene signatures to improve the prognosis of PCa urgently need to be developed. To explore the prognostic value of m5C regulators, a binary classifier based on the m5C score was built in our research. PCa patients with higher m5C scores had a higher likelihood of BCR, and the robustness of this score was verified in four external cohorts.

Endocrine therapy and chemotherapy have been the cornerstones of PCa treatment in the last decade. According to the PAM50 classification scheme, PCa patients with LumB subtype have a worse prognosis than those with the basal and LumA subtypes, but LumB patients benefit more from androgen-deprivation therapy (ADT) [34]. LumA tumors have been shown to be more sensitive to gemcitabine, and basal tumors have been shown to be more sensitive to docetaxel [35, 36]. Moreover, luminal tumors treated with an ARSI have significantly better survival than basal tumors. In addition, the higher m5C score group had a poorer prognosis in every PAM50 subtype. This result indicates that patients with higher m5C scores in each subtype should receive more active intervention with drugs to which they are sensitive. On the other hand, the m5C score can predict the clinical response to and outcome of ARSI. Our results suggest that the benefit of ARSI treatment is more remarkable in a those with lower m5C scores. Moreover, the high m5C group was sensitive to the apoptosis-regulating drug venetoclax, as well as the kinase inhibitor ruxolitinib, based on the Genomics of Drug Sensitivity in Cancer (GDSC) analysis. In general, our results suggest the novel possibility of making precise decisions regarding endocrine therapy and chemotherapy in PCa.

In many malignancies, cancer immunotherapy has been shown to be a successful and crucial treatment option. In this manuscript, we showed that the DEGs between the m5C score groups were significantly enriched in PD-L1 expression and the PD-1 checkpoint pathway. The m5C score was found to be positively related to immunomodulators, such as TGFB1, CXCL10, GZMA, PD-1, CTLA4, IDO1, HAVCR2, and LAG3, which are critical for the clinical benefit of immunotherapy. Similarly, the high m5C score group was correlated with poor clinical benefit of PD1/PDL1 inhibitor therapy in three different cancers. Overall, the m5C score may predict the response to immunotherapy in PCa. However, it should be noted that the relationship between the m5C score and the immune TME is complicated, and our study lacked samples from PCa patients who received immunotherapy. These factors could be discussed in further studies.

The implementation of the m5C scoring system based on the expression of seven molecules in clinical practice may face significant challenges, particularly in terms of economics. To address this, ALYREF, the hub gene of the m5C score, could be used as a cost-effective alternative. Our study also demonstrated that ALYREF promotes PCa progression through in silico and experimental validations. Therefore, in exploring the potential clinical applications of AYREF in the future, detection of RNA expression in samples or immunohistochemical detection of protein levels are both viable options. Furthermore, our findings indicate that ALYREF is expressed in luminal epithelial and neuroendocrine epithelial cells. The heterogeneity of cancer refers to the presence of variable populations of cells with distinct genomic signatures within tumors or at different sites within the same patient. Approximately 25% of tumors in men who die of PCa are neuroendocrine tumors, which have been shown to be associated with poor prognosis.

## Conclusions

In conclusion, this study offers a new m5C-related predictor for determining prognosis and the response to multiple therapies in PCa. Our study provides both a prognostic biomarker and a potential therapeutic target for identifying and improving the prognoses of patients with different tumor molecular subtypes, predicting patient therapy response, and enhancing personalized therapy.

## Supplementary Information

**Additional file 1: Table S1.** Public datasets in this study. **Table S2.** Oligo sequence of siRNAs targeting ALYREF transcript. **Table S3.** Single cell sequencing expression profile. **Table S4.** COX & network of m5C regulators. **Table S5.** Cluster KEGG, and GO. **Table S6.** Coefficient. **Table S7.** m5C scores KEGG & GO. **Table S8.** GDSC. **Table S9.** ssGSEA & cibersort. **Table S10.** Tissue Microarray. **Table S11.** siALYREF RNA-seq. **Table S12.** ALYREF RIP-seq. **Table S13.** ALYREF Functional Enrichment

**Additional file 2: Figure S1.** The overall study design and the workflow of the research. **Figure S2.** The m5C regulators’ expression landscape and their prognosis value. (A) The differential gene expression of the 13 m5C regulators in the MSI and non-MSI subgroups in the TCGA-PRAD cohort. *P < 0.05; **P < 0.01; ***P < 0.001. (B) The differential gene expression of the 13 m5C regulators in the TP53_wt and TP53_mut subgroups in the TCGA-PRAD cohort. (C) The differential gene expression of the 13 m5C regulators in the ERG-fusion and non-ERG-fusion subgroups in the TCGA-PRAD cohort. (D) The Kaplan–Meier survival curves show five m5C genes to be favorable factors. Log-rank test (TRDMT, P=0.001; ALKBH1, P=0.002; NSUN7, P=0.004; TET1, P=0.001; NSUN4, P=0.034). (E) The Kaplan–Meier curves demonstrate another five m5C genes to be risk factors. Log-rank test (YBX1, P=0.002; NSUN2, P=0.014; NSUN5, P<0.001; NOP2, P=0.036; ALYREF, P<0.001). **Figure S3.** Depicting the 13 m5C regulator genes in prostatic cells with t-distributed stochastic neighbor embedding (t-SNE) and uniform manifold approximation and projection (UMAP). **Figure S4.** The unsupervised clustering of m5C regulators in the TCGA-PRAD cohort and prognosis differences under several scenarios of m5C cluster grouping. (A) BIC plot for models fitted. (B- F) The Kaplan–Meier curves of different cluster numbers of grouping. Log-rank test (k=2, P=0.00069; k=3, P=0.0017; k=4, P<0.0001; k=5, P<0.0001; k=6, P=0.00056). **Figure S5.** The clinical relevance of the binary m5C clustering and the functional annotations for differentially expressed genes between the two m5C clusters. (A) The proportion of patients’ clinical factors in the two m5C clusters in the TCGA-PRAD cohort. (B & C) The GO and KEGG functional annotations for DEGs between the two m5C clusters. **Figure S6.** Construction of the m5C score system and the nomogram. (A) Selection of the optimal parameter (lambda) in the LASSO model. (B) LASSO coefficient profiles of the 13 m5C regulator genes. A coefficient profile plot was generated against the log (lambda) sequence. (C) The nomogram to predict BCR-free survival of PCa patients in the TCGA-PRAD cohort and the calibration plot for the 1-year (blue line) and 5-year (red line) BCR free survival predictions. **Figure S7.** The correlation of m5C clusters and m5C score, and GSVA enrichment of m5C scores. (A) The gene expression differences of m5C regulators in PCa patients within low- and high- m5C score subgroups using the TCGA-PRAD database (P < 0.05; **P < 0.01; ***P < 0.001). (B) The Sankey diagram shows correlations between m5C clusters and m5C score and BCR status. (C) The m5C score difference between 2 m5C clusters. (D) The heatmap shows the GSVA score distribution of representative pathways curated from the MSigDB database in two m5C score subgroups. **Figure S8.** The function annotations and mutation landscape of the m5C score subgroups in the TCGA-PRAD cohort. (A & B) GO and KEGG function annotations of DEGs between the two m5C score subgroups. (C & D) Mutation landscapes of significantly mutated genes in TCGA-PRAD in low- and high- m5C score subgroups, respectively. Each column represents one patient with gene mutations. The top bar plot shows tumor mutation load, and the right horizontal bar plot shows the mutation signatures of each gene in mutated samples of each m5C score subgroup. **Figure S9.** The molecular signatures relevance of the m5C score in the ARSI cohort. (A-D) Figures 1-4 show the correlations between m5C score and CCP score, m5C score and RB-loss score, m5C score and NEPC score, and m5C score and AR score, respectively, in the ARSI cohort. R represents Pearson’s correlation coefficient, and P stands for P-value (ns, P ≥ 0.05; *P < 0.05; **P < 0.01; ***P < 0.001). The m5C score positively correlated with both CCP score (A) and RB-loss score (B). **Figure S10.** The immune landscape of the m5C score groups in TCGA-PRAD cohort. (A & B) The fraction variation of tumor-infiltrating immune cells in two m5C score groups using the ssGSEA (A) and CIBERSORT (B) algorithms, respectively. Within each group, the scattered dot represents the TME cell expression value. The thick line represents the median value (*P < 0.05; **P < 0.01; ***P < 0.001). (C & D) The heatmap shows the correlations between the m5C score grouping and the clinicopathological characters and between the m5C score grouping and the score of tumor-infiltrating immune cells, using the ssGSEA (C) algorithm and CIBERSORT (D) algorithm, respectively. **Figure S11. **The correlation between TME subtypes and the m5C score, cytokine and chemokine milieu among m5C score groups. (A) The differences in the m5C score between four PCa TME subtypes in TCGA-PRAD cohort. (B) The expression levels of macrophage polarization-related genes (M2 marker genes, IL1R1, FIZ1, TGFB1, IL10, ARG1, CD163; M1 marker genes, NOS2, IL23A, IL15RA, IL12A). (C) The expression levels of immune-checkpoint-relevant genes (IDO1, CD274, HAVCR2, PDCD1, CTLA4, LAG3, and PDCD1LG2) in the m5C score groups. (D) The expression levels of immune activation-relevant genes (CXCL10, CXCL9, GZMA, GZMB, PRF1, CD8A, IFNG, TBX2, and TNF) in three Gene-cluster groups. Within each group, the scattered dots represent gene values. The thick line represents the median value. The bottom and top of the boxes are the 25th and 75th percentiles (interquartile range). The whiskers encompass 1.5 times the interquartile range. The range of P-value is labeled above each boxplot with asterisks. (*P < 0.05, **P < 0.01, ***P < 0.001). **Figure S12.** ALYREF promotes prostate cancer progression. (A) Western blotting validated the knockdown efficacy of siRNAs (si-392, si-615, si-767) targeting ALYREF in the LNCaP cell line. β-actin was the loading control. (B) Transwell assay confirmed silencing ALYREF diminished LNCaP cell invasion ability. (C) 4-day CCK-8 assay reflected the knockdown of ALYREF attenuated LNCaP cell proliferation. (D) Plate colony formation assay showed silencing ALYREF downregulated C4-2 cell colony formation ability. (E) ALYREF’s pan-cancer prognosis analysis (progression-free survival, PFS), including 10 types of cancers (KICH, kidney chromophobe; PRAD, prostate adenocarcinoma; UVM, uveal melanoma; KIRP, kidney renal papillary cell carcinoma; ACC,adrenocortical carcinoma; GBMLGG, glioma; LGG, brain lower grade glioma; LIHC, liver hepatocellular carcinoma; KIPAN,pan-kidney cohort (KICH+KIRC+KIRP); OV, ovarian serous cystadenocarcinoma) Error bar indicates mean ± SD. *P <0.05, **P < 0.01, ****P < 0.0001. **Figure S13.** RNA-seq and RIP-seq co-analysis of ALYREF. (A)Venn diagram showed 376 overlapped genes between si-ALYREF RNA-seq data and ALYREF RIP-seq data. (B)The top 15 GO terms in the functional enrichment analysis of RNA-seq and RIP-seq co-analysis. (C)The top 15 KEGG pathways in the functional enrichment analysis RNA-seq and RIP-seq co-analysis

## Data Availability

The datasets used and/or analyzed during the current study are available within the manuscript and its supplementary information files.

## Abbreviations

m5C: 5-Methylcytosine
ARSI: Androgen receptor signaling inhibitor
BCR: Biochemical recurrence
CNV: Copy number variation
CCP: Cycle progression
D: Depleted
DEGs: Differentially expressed genes
DNMT2: DNA methyltransferase member 2
F: Fibrotic
GEO: Gene Expression Omnibus
GO: Gene ontology
GDSC: Genomics of Drug Sensitivity in Cancer
ICB: Immune checkpoint blockade
IE: Immune-enriched
IE/F: Immune-enriched with fibrotic
IHC: Immunohistochemistry
KEGG: Kyoto Encyclopedia of Genes and Genomes
LASSO: Least absolute shrinkage and selection operator
LumA: Luminal A
LumB: Luminal B
MDSCs: Myeloid-derived suppressor cells
NSCLC: Non-small cell lung cancer
PD-L1: Programmed Cell Death Ligand 1
PD-1: Programmed cell death protein 1
PFI: Progression-free interval
PRAD: Prostate adenocarcinoma
PCa: Prostate adenocarcinoma
