# Supplementary material for: Identification and verification of an ALYREF-involved 5-methylcytosine based signature for stratification of prostate cancer patients and prediction of clinical outcome and response to therapies
Source: Discov Oncol. 2023 May 8;14:62. doi: 10.1007/s12672-023-00671-w (PMC10167087; doi:10.1007/s12672-023-00671-w)
Supplement: Supplementary file 2 — Additional file 2: Figure S1. The overall study design and the workflow of the research. Figure S2. The m5C regulators’ expression landscape and their prognosis value. (A) The differential gene expression of the 13 m5C regulators in the MSI and non-MSI subgroups in the TCGA-PRAD cohort. *P < 0.05; **P < 0.01; ***P < 0.001. (B) The differential gene expression of the 13 m5C regulators in the TP53_wt and TP53_mut subgroups in the TCGA-PRAD cohort. (C) The differential gene expression of the 13 m5C regulators in the ERG-fusion and non-ERG-fusion subgroups in the TCGA-PRAD cohort. (D) The Kaplan–Meier survival curves show five m5C genes to be favorable factors. Log-rank test (TRDMT, P=0.001; ALKBH1, P=0.002; NSUN7, P=0.004; TET1, P=0.001; NSUN4, P=0.034). (E) The Kaplan–Meier curves demonstrate another five m5C genes to be risk factors. Log-rank test (YBX1, P=0.002; NSUN2, P=0.014; NSUN5, P<0.001; NOP2, P=0.036; ALYREF, P<0.001). Figure S3. Depicting the 13 m5C regulator genes in prostatic cells with t-distributed stochastic neighbor embedding (t-SNE) and uniform manifold approximation and projection (UMAP). Figure S4. The unsupervised clustering of m5C regulators in the TCGA-PRAD cohort and prognosis differences under several scenarios of m5C cluster grouping. (A) BIC plot for models fitted. (B- F) The Kaplan–Meier curves of different cluster numbers of grouping. Log-rank test (k=2, P=0.00069; k=3, P=0.0017; k=4, P<0.0001; k=5, P<0.0001; k=6, P=0.00056). Figure S5. The clinical relevance of the binary m5C clustering and the functional annotations for differentially expressed genes between the two m5C clusters. (A) The proportion of patients’ clinical factors in the two m5C clusters in the TCGA-PRAD cohort. (B & C) The GO and KEGG functional annotations for DEGs between the two m5C clusters. Figure S6. Construction of the m5C score system and the nomogram. (A) Selection of the optimal parameter (lambda) in the LASSO model. (B) LASSO coefficient profiles of the 13 m5C re [file 12672_2023_671_MOESM2_ESM.pptx]

## Slide 1
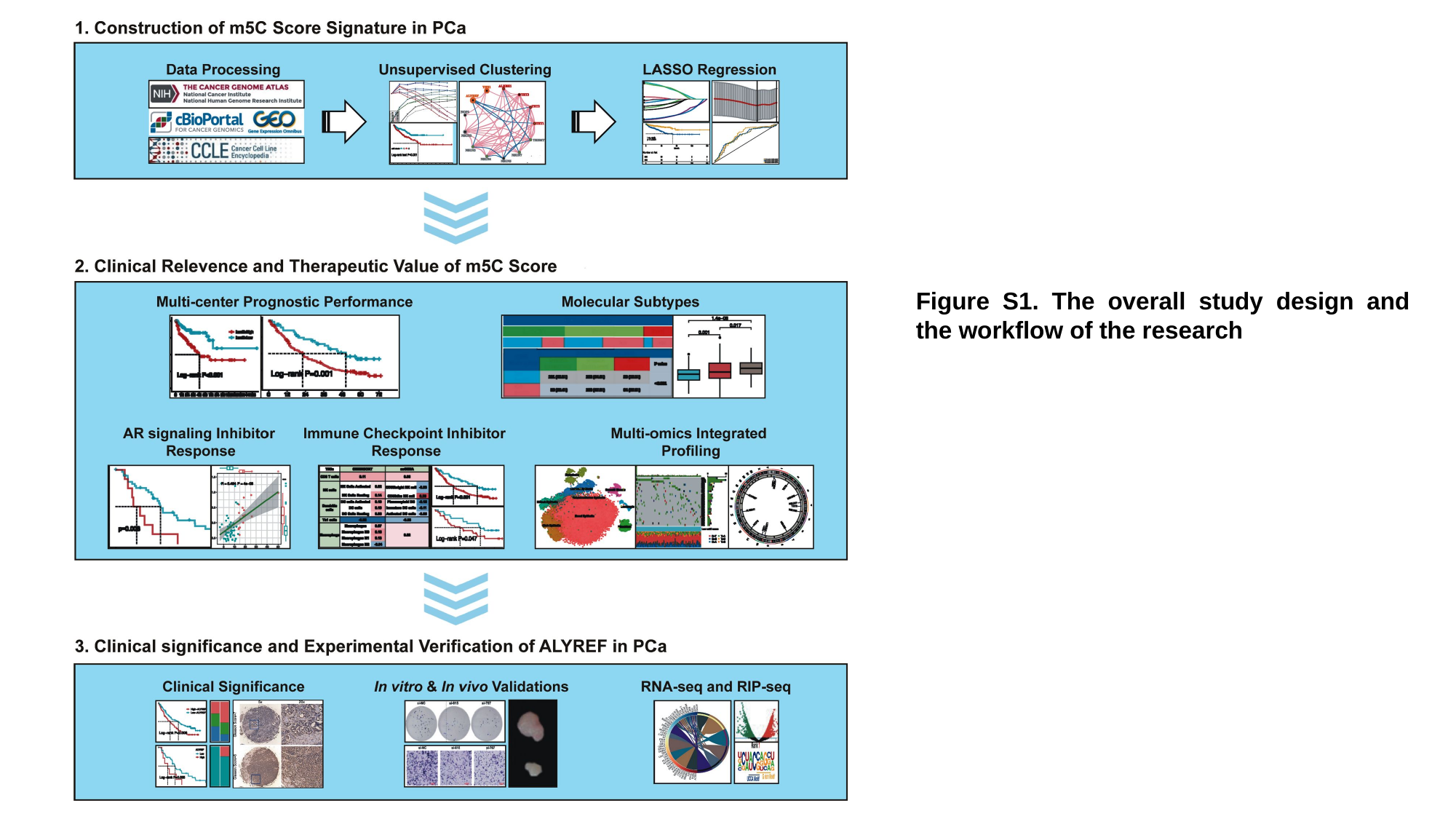

Figure S1. The overall study design and the workflow of the research

## Slide 2
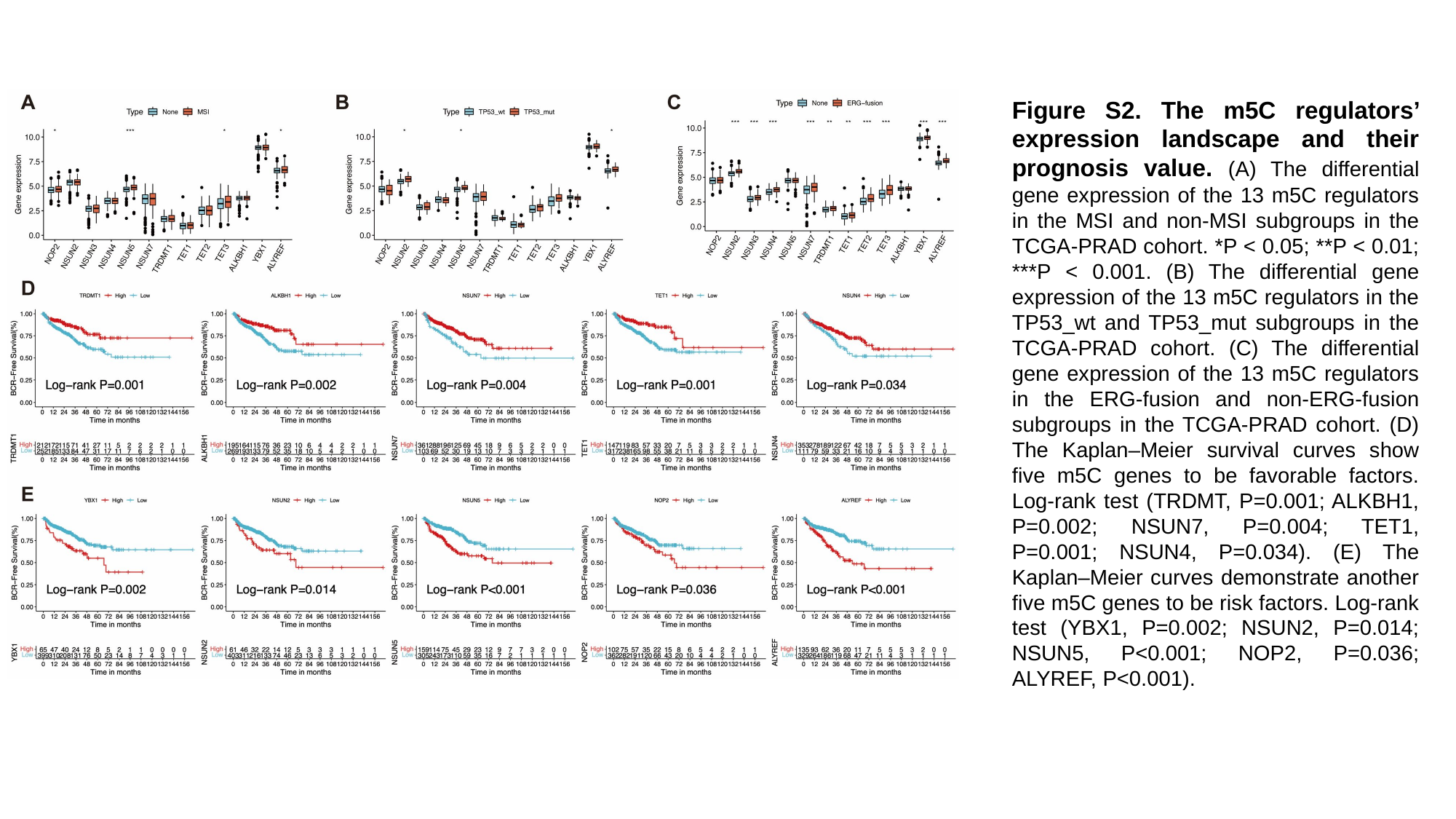

Figure S2. The m5C regulators’ expression landscape and their prognosis value. (A) The differential gene expression of the 13 m5C regulators in the MSI and non-MSI subgroups in the TCGA-PRAD cohort. *P < 0.05; **P < 0.01; ***P < 0.001. (B) The differential gene expression of the 13 m5C regulators in the TP53_wt and TP53_mut subgroups in the TCGA-PRAD cohort. (C) The differential gene expression of the 13 m5C regulators in the ERG-fusion and non-ERG-fusion subgroups in the TCGA-PRAD cohort. (D) The Kaplan–Meier survival curves show five m5C genes to be favorable factors. Log-rank test (TRDMT, P=0.001; ALKBH1, P=0.002; NSUN7, P=0.004; TET1, P=0.001; NSUN4, P=0.034). (E) The Kaplan–Meier curves demonstrate another five m5C genes to be risk factors. Log-rank test (YBX1, P=0.002; NSUN2, P=0.014; NSUN5, P<0.001; NOP2, P=0.036; ALYREF, P<0.001).

## Slide 3
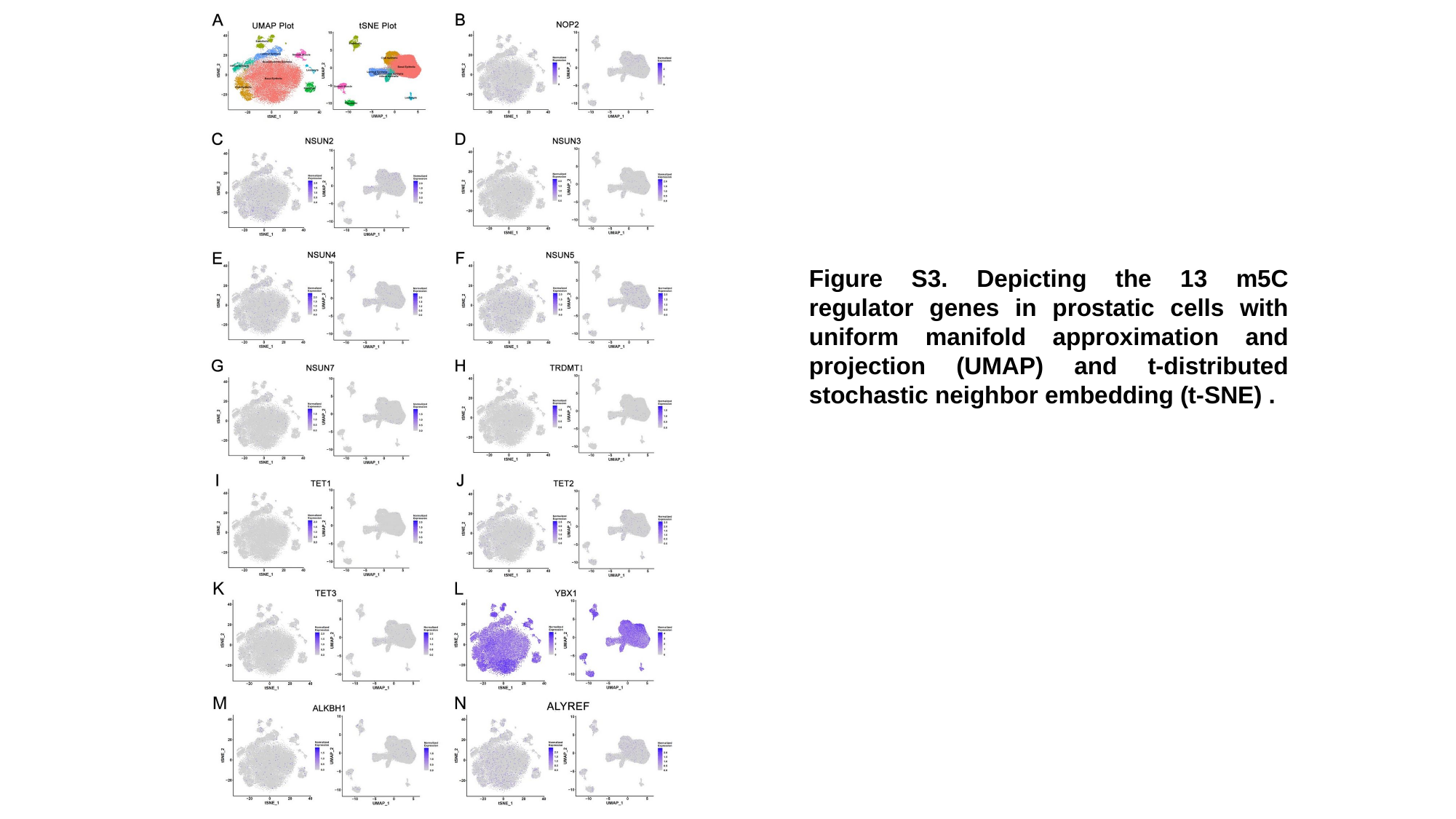

Figure S3. Depicting the 13 m5C regulator genes in prostatic cells with uniform manifold approximation and projection (UMAP) and t-distributed stochastic neighbor embedding (t-SNE) .

## Slide 4
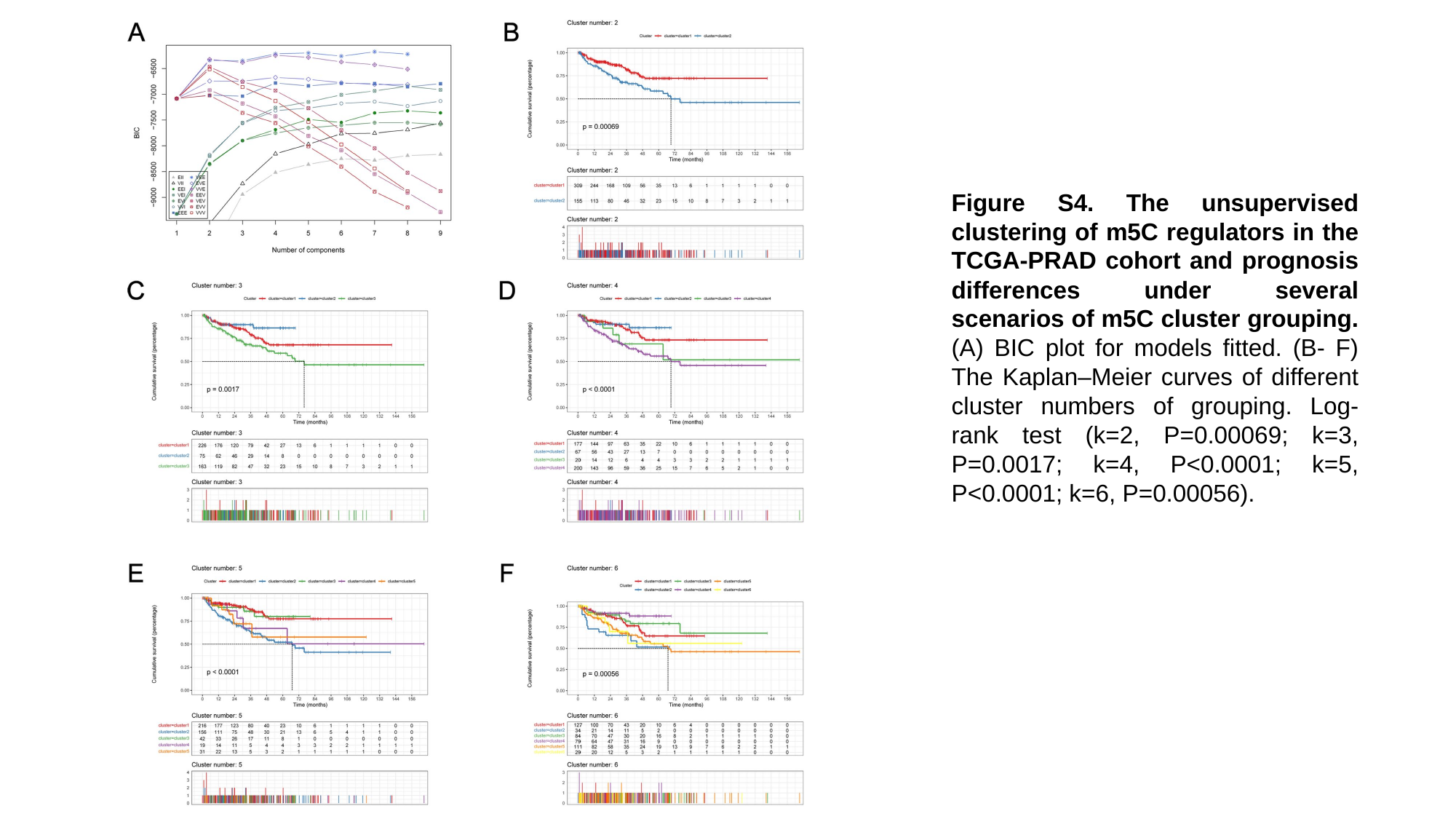

Figure S4. The unsupervised clustering of m5C regulators in the TCGA-PRAD cohort and prognosis differences under several scenarios of m5C cluster grouping. (A) BIC plot for models fitted. (B- F) The Kaplan–Meier curves of different cluster numbers of grouping. Log-rank test (k=2, P=0.00069; k=3, P=0.0017; k=4, P<0.0001; k=5, P<0.0001; k=6, P=0.00056).

## Slide 5
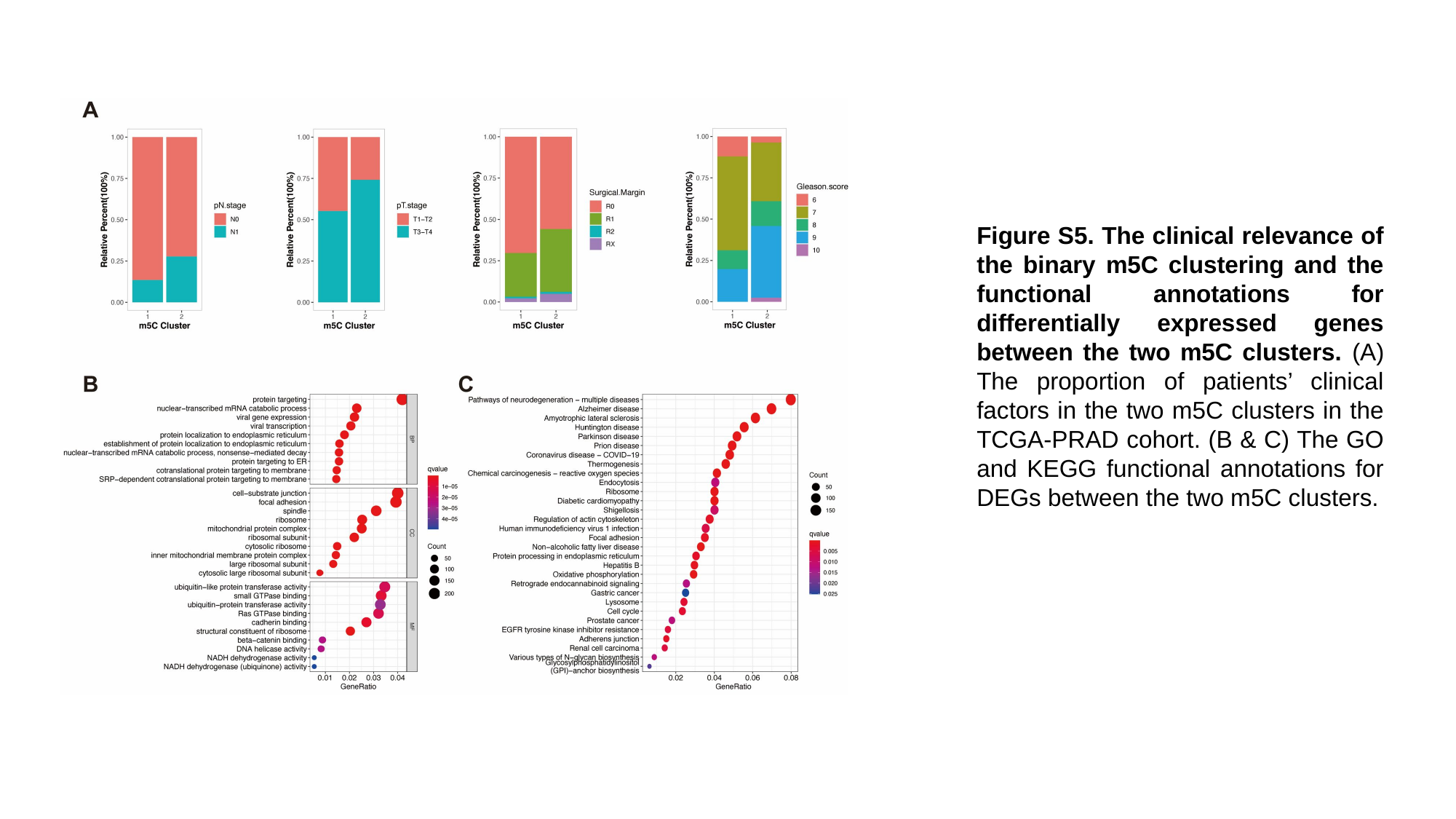

Figure S5. The clinical relevance of the binary m5C clustering and the functional annotations for differentially expressed genes between the two m5C clusters. (A) The proportion of patients’ clinical factors in the two m5C clusters in the TCGA-PRAD cohort. (B & C) The GO and KEGG functional annotations for DEGs between the two m5C clusters.

## Slide 6
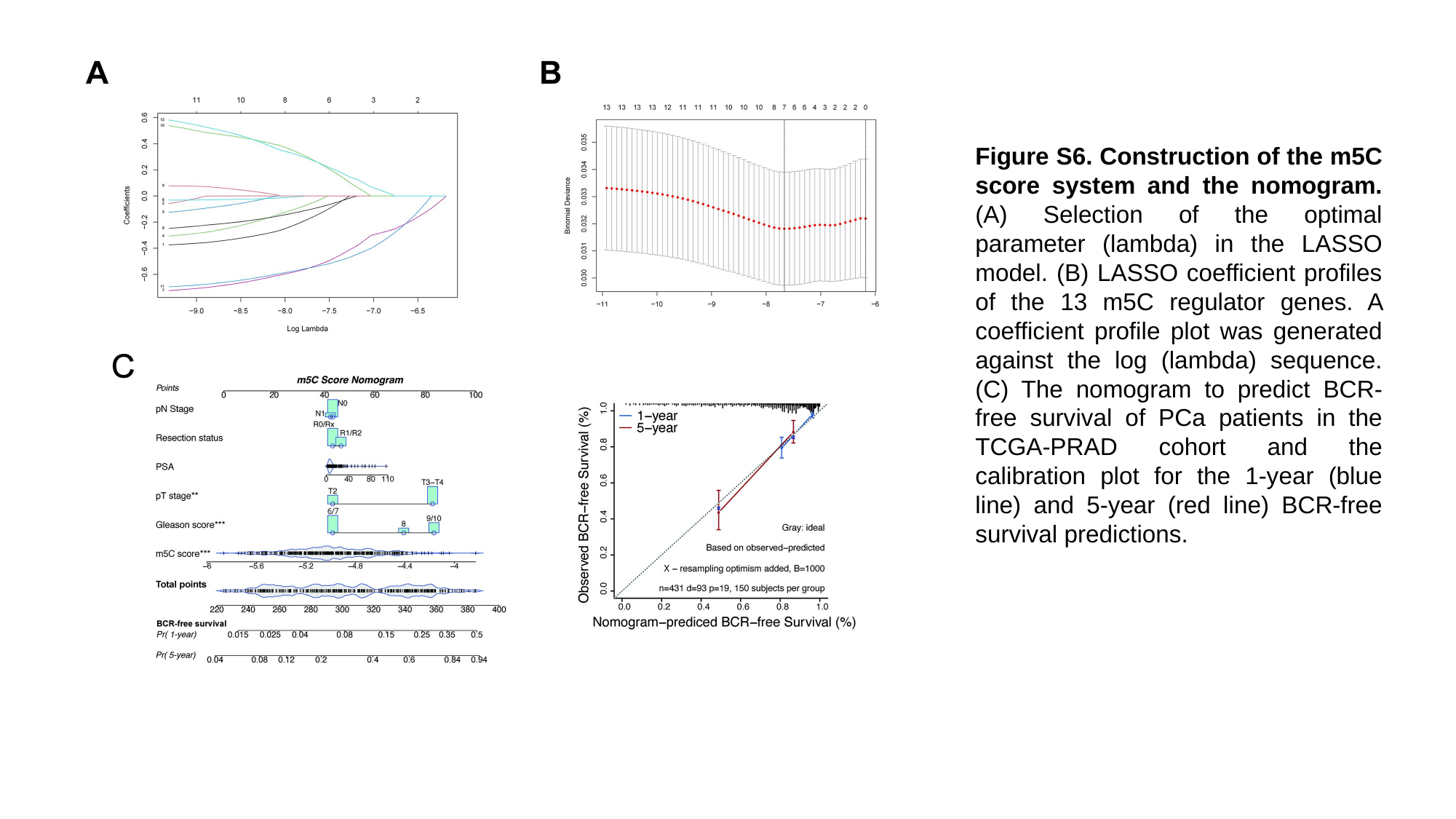

Figure S6. Construction of the m5C score system and the nomogram. (A) Selection of the optimal parameter (lambda) in the LASSO model. (B) LASSO coefficient profiles of the 13 m5C regulator genes. A coefficient profile plot was generated against the log (lambda) sequence. (C) The nomogram to predict BCR-free survival of PCa patients in the TCGA-PRAD cohort and the calibration plot for the 1-year (blue line) and 5-year (red line) BCR-free survival predictions.

## Slide 7
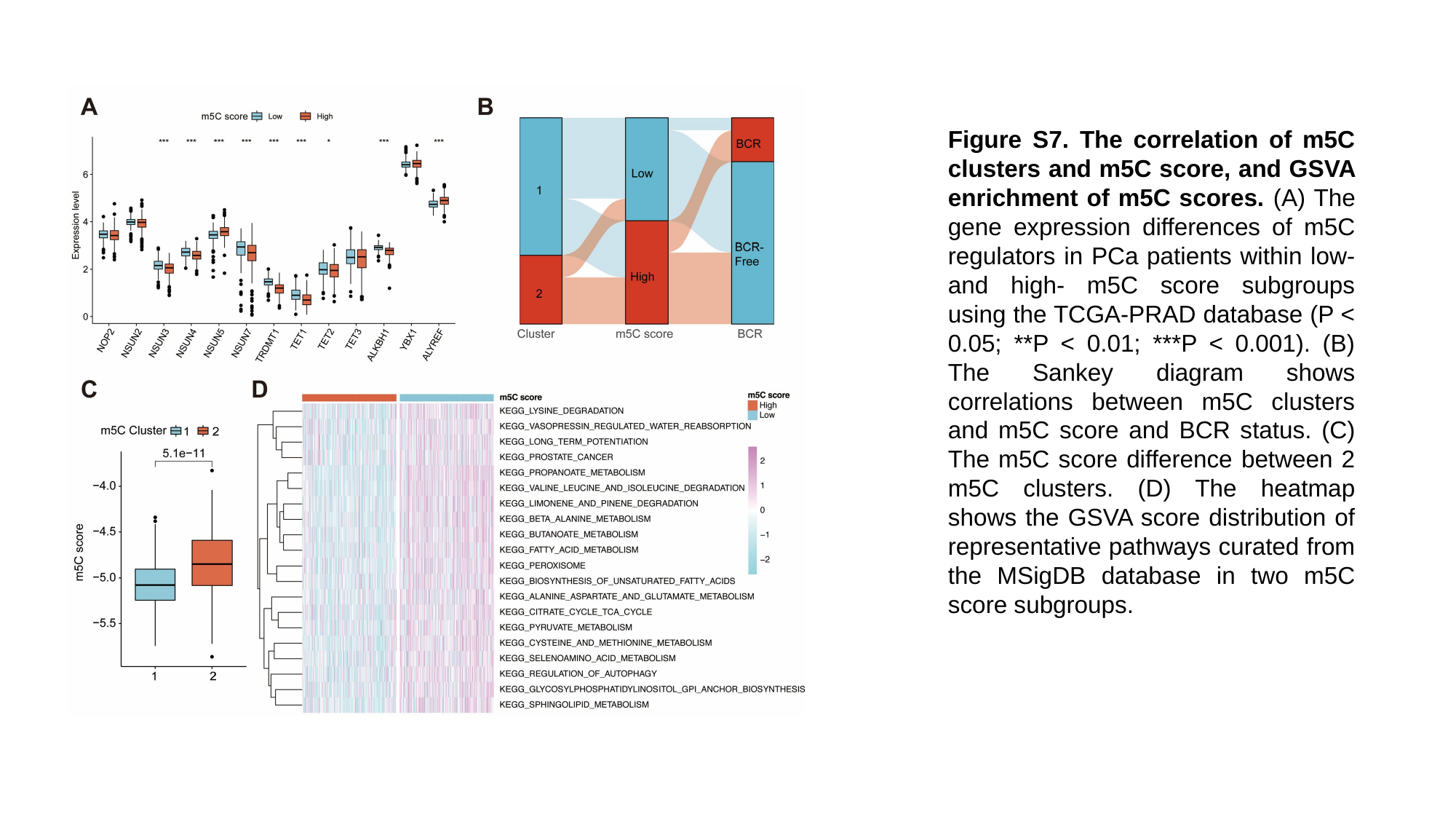

Figure S7. The correlation of m5C clusters and m5C score, and GSVA enrichment of m5C scores. (A) The gene expression differences of m5C regulators in PCa patients within low- and high- m5C score subgroups using the TCGA-PRAD database (P < 0.05; **P < 0.01; ***P < 0.001). (B) The Sankey diagram shows correlations between m5C clusters and m5C score and BCR status. (C) The m5C score difference between 2 m5C clusters. (D) The heatmap shows the GSVA score distribution of representative pathways curated from the MSigDB database in two m5C score subgroups.

## Slide 8
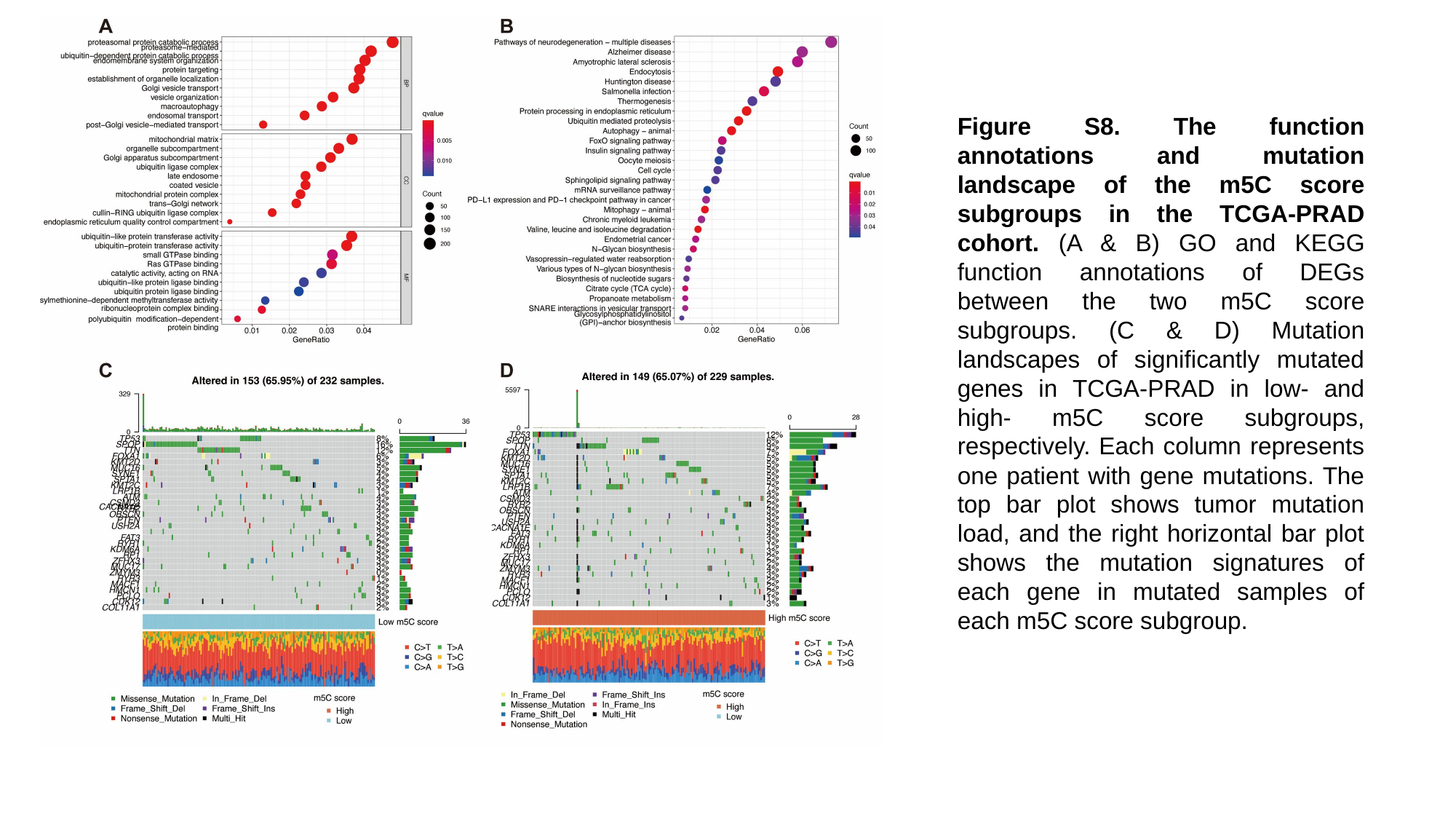

Figure S8. The function annotations and mutation landscape of the m5C score subgroups in the TCGA-PRAD cohort. (A & B) GO and KEGG function annotations of DEGs between the two m5C score subgroups. (C & D) Mutation landscapes of significantly mutated genes in TCGA-PRAD in low- and high- m5C score subgroups, respectively. Each column represents one patient with gene mutations. The top bar plot shows tumor mutation load, and the right horizontal bar plot shows the mutation signatures of each gene in mutated samples of each m5C score subgroup.

## Slide 9
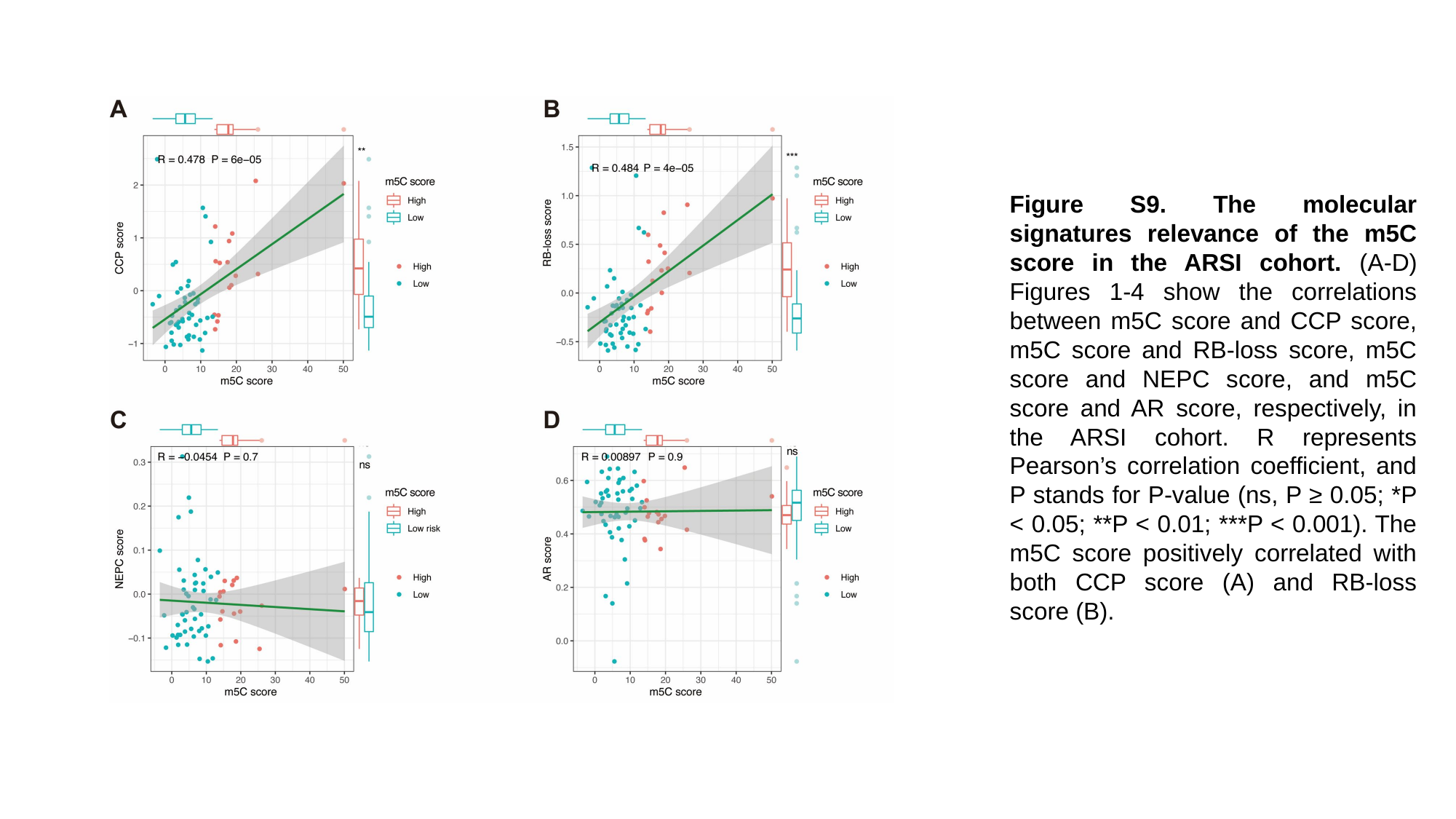

Figure S9. The molecular signatures relevance of the m5C score in the ARSI cohort. (A-D) Figures 1-4 show the correlations between m5C score and CCP score, m5C score and RB-loss score, m5C score and NEPC score, and m5C score and AR score, respectively, in the ARSI cohort. R represents Pearson’s correlation coefficient, and P stands for P-value (ns, P ≥ 0.05; *P < 0.05; **P < 0.01; ***P < 0.001). The m5C score positively correlated with both CCP score (A) and RB-loss score (B).

## Slide 10
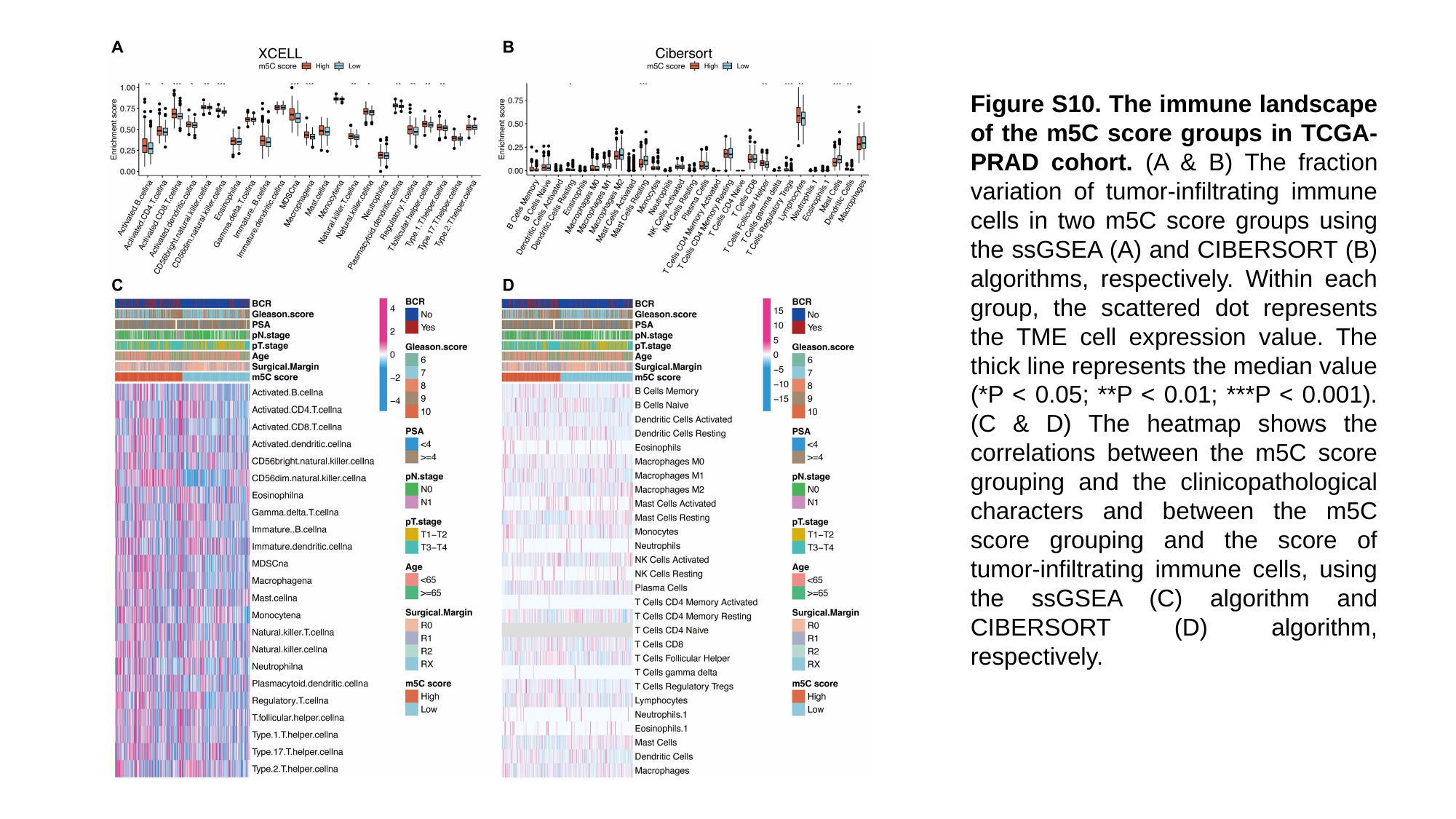

Figure S10. The immune landscape of the m5C score groups in TCGA-PRAD cohort. (A & B) The fraction variation of tumor-infiltrating immune cells in two m5C score groups using the ssGSEA (A) and CIBERSORT (B) algorithms, respectively. Within each group, the scattered dot represents the TME cell expression value. The thick line represents the median value (*P < 0.05; **P < 0.01; ***P < 0.001). (C & D) The heatmap shows the correlations between the m5C score grouping and the clinicopathological characters and between the m5C score grouping and the score of tumor-infiltrating immune cells, using the ssGSEA (C) algorithm and CIBERSORT (D) algorithm, respectively.

## Slide 11
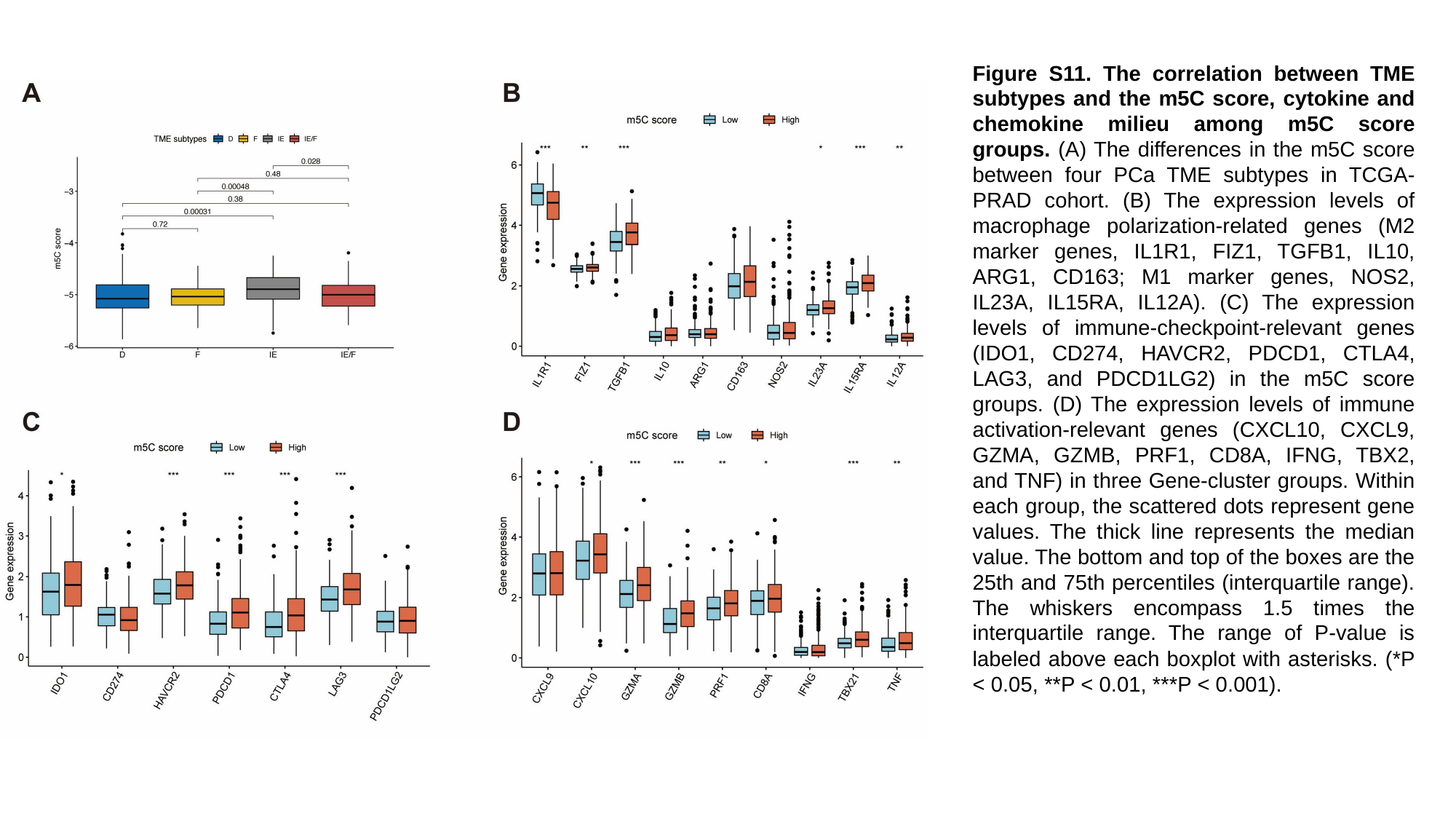

Figure S11. The correlation between TME subtypes and the m5C score, cytokine and chemokine milieu among m5C score groups. (A) The differences in the m5C score between four PCa TME subtypes in TCGA-PRAD cohort. (B) The expression levels of macrophage polarization-related genes (M2 marker genes, IL1R1, FIZ1, TGFB1, IL10, ARG1, CD163; M1 marker genes, NOS2, IL23A, IL15RA, IL12A). (C) The expression levels of immune-checkpoint-relevant genes (IDO1, CD274, HAVCR2, PDCD1, CTLA4, LAG3, and PDCD1LG2) in the m5C score groups. (D) The expression levels of immune activation-relevant genes (CXCL10, CXCL9, GZMA, GZMB, PRF1, CD8A, IFNG, TBX2, and TNF) in three Gene-cluster groups. Within each group, the scattered dots represent gene values. The thick line represents the median value. The bottom and top of the boxes are the 25th and 75th percentiles (interquartile range). The whiskers encompass 1.5 times the interquartile range. The range of P-value is labeled above each boxplot with asterisks. (*P < 0.05, **P < 0.01, ***P < 0.001).

## Slide 12
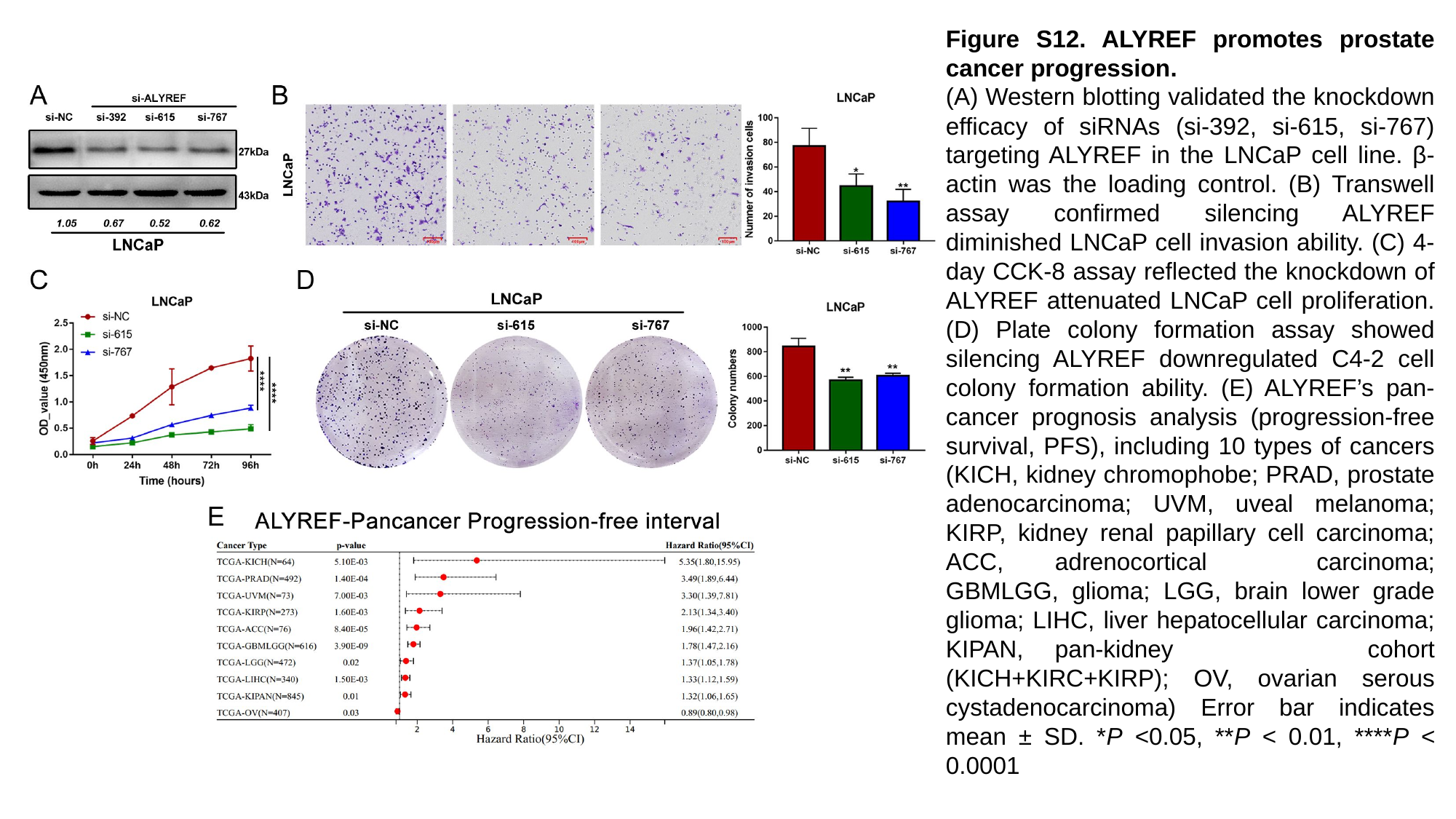

Figure S12. ALYREF promotes prostate cancer progression.
(A) Western blotting validated the knockdown efficacy of siRNAs (si-392, si-615, si-767) targeting ALYREF in the LNCaP cell line. β-actin was the loading control. (B) Transwell assay confirmed silencing ALYREF diminished LNCaP cell invasion ability. (C) 4-day CCK-8 assay reflected the knockdown of ALYREF attenuated LNCaP cell proliferation. (D) Plate colony formation assay showed silencing ALYREF downregulated C4-2 cell colony formation ability. (E) ALYREF’s pan-cancer prognosis analysis (progression-free survival, PFS), including 10 types of cancers (KICH, kidney chromophobe; PRAD, prostate adenocarcinoma; UVM, uveal melanoma; KIRP, kidney renal papillary cell carcinoma; ACC,	adrenocortical carcinoma; GBMLGG, glioma; LGG, brain lower grade glioma; LIHC, liver hepatocellular carcinoma; KIPAN,	pan-kidney cohort (KICH+KIRC+KIRP); OV, ovarian serous cystadenocarcinoma) Error bar indicates mean ± SD. *P <0.05, **P < 0.01, ****P < 0.0001

## Slide 13
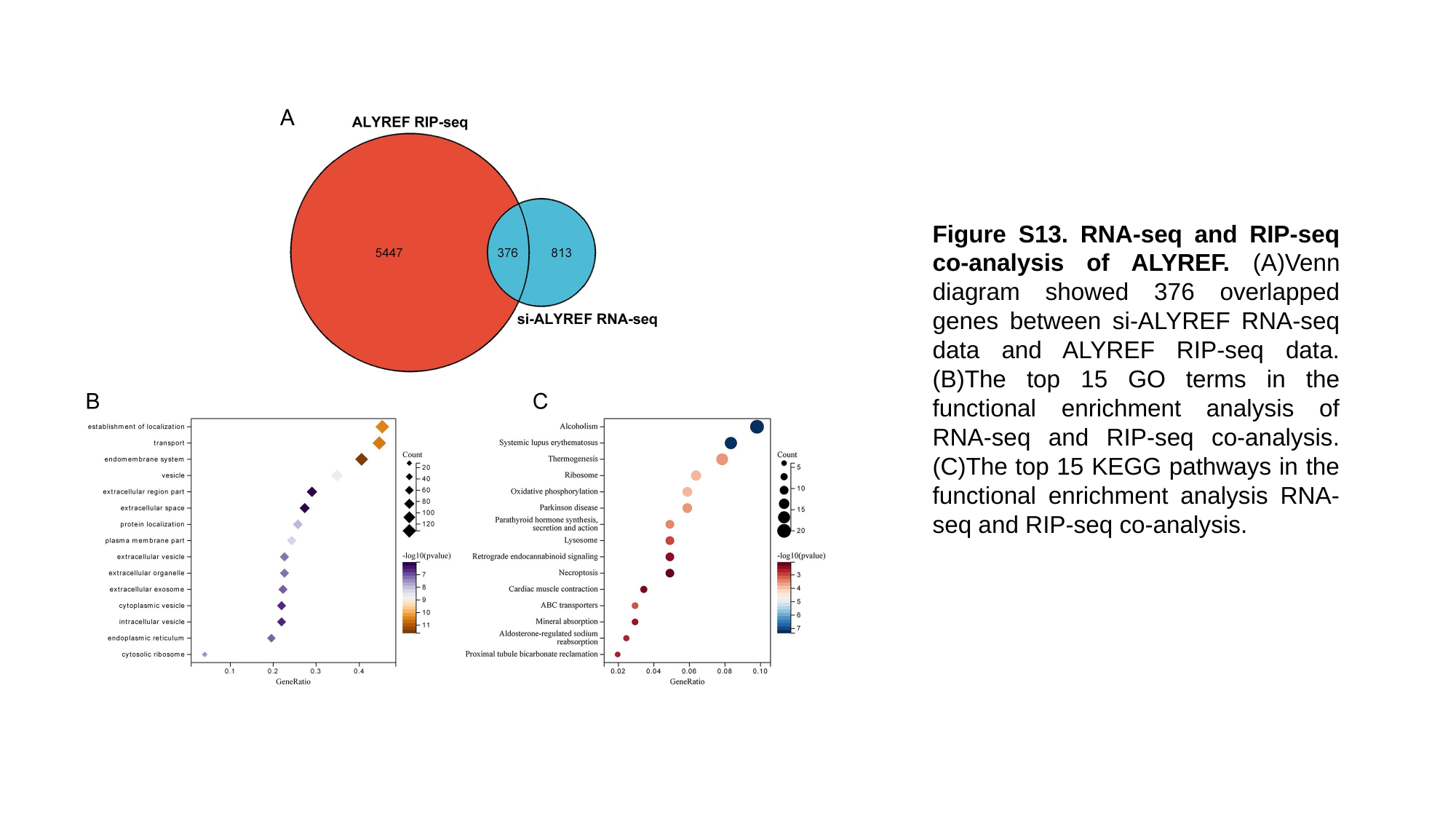

Figure S13. RNA-seq and RIP-seq co-analysis of ALYREF. (A)Venn diagram showed 376 overlapped genes between si-ALYREF RNA-seq data and ALYREF RIP-seq data. (B)The top 15 GO terms in the functional enrichment analysis of RNA-seq and RIP-seq co-analysis. (C)The top 15 KEGG pathways in the functional enrichment analysis RNA-seq and RIP-seq co-analysis.
